# An extended best–worst multiple reference point method: application in the assessment of non-life insurance companies

**DOI:** 10.1007/s12351-022-00731-z

**Published:** 2022-09-10

**Authors:** Amelia Bilbao-Terol, Mar Arenas-Parra, Raquel Quiroga-García, Celia Bilbao-Terol

**Affiliations:** 1 Faculty of Commerce Tourism and Social Sciences Jovellanos, University of Oviedo, Laboral Ciudad de La Cultura, 33203 Gijón, CP Spain; 2 Faculty of Business and Economics, University of Oviedo, Avda. Del Cristo s/n, Oviedo, Spain

**Keywords:** Multi-criteria decision making, Interval value, Pairwise comparison, Non-life insurance company, Best–worst method, Multiple reference point method

## Abstract

In this paper a multi-criteria decision-making (MCDM) method is developed to rank a set of insurance companies. The proposed method is based on combining two MCDM methods: Extended Best–Worst (EBW) and Multiple Reference Point (MRP) methods. We formulate the problem of finding a priority vector from a set of interval pairwise comparisons applying an EBW method which allows the decision maker (DM) to use interval values in order to describe the relative importance of one criterion over another. The EBW method, using fuzzy set theory, can successfully handle the vagueness and ambiguity present in the judgments. Lastly, the MRP method is employed to obtain an overall score for each company using the weights established at the first stage. A case study is presented to rank Spanish non-life insurance companies based on the constructed model. Since the evaluation of insurance companies involves a great number of indicators, it is a complex MCDM issue. The results show the effectiveness of the proposed method and offer an insightful reference for an evaluation of the insurance industry.

## Introduction

The insurance market plays a key role in the financial markets and the economy of any country. Insurers provide security to companies and individuals who transfer their risks to them in exchange for the payment of a premium (Rejikumar et al. 2019). At the same time, insurance companies collect the premiums and invest them in the financial markets contributing in this way to economic development (Akyüz et al. 2020). Thus, insurance functions can be resumed in assurance and intermediation.

Assurance is the main service provided by insurers. It is related to risk-pooling and risk bearing services, as well as to the “real” financial service (Cummins and Nini 2002; Cummins and Rubio-Misas 2006; Leverty and Grace 2010). Insurers use the premiums received to pay claims. For this purpose, they incur actuarial and subscription expenses that, together with the capital reserve for extraordinary claims, allow the creation of value added. Real financial service is related to financial advisory services, risk management or loss prevention. Within the intermediation function, insurers obtain funds from their policyholders through premiums and invest them in financial markets in order to guarantee the payment of claims when they occur. This service is especially relevant in life insurance, where the moment of collection of the premiums and the payment of the claim are, normally, separated in time. Furthermore, this type of insurance guarantees an additional interest.

The competitiveness of insurance companies relies on their ability to maximise profits and improve the quality of their services. That is why the efficiency of insurance companies has long been studied in the literature (see, e.g., Eling and Luhnen 2010; Cummins and Weiss 2013 for a review). Efficiency measures the ability of companies to obtain the maximum output with a given amount of input, or conversely, use the least amount of inputs to achieve a certain level of output. The models to study efficiency are the non-parametric Data Envelopment Analysis (DEA) model and the parametric Stochastic Frontier Analysis (SFA) model. Both models in their different variations have been applied to the insurance market and allow a ranking of companies according to their level of efficiency. This allows companies to know their positions relative to their competitors. But although efficiency and profitability are usually related, only considering efficiency suffers the weakness that it does not guarantee profitability (Ventakateswarlu et al. 2016), nor financial strength (Eckles and Pottier 2011).

Meanwhile, the profitability of a company is related to its ability to make its income exceed its expenses and it can be measured through financial ratios which are widely used in the literature. It is related to its financial strength, given that better financial health is interpreted as presenting a greater likelihood that the company will meet all its obligations, thereby bearing a lower risk of insolvency and a greater likelihood of profit (Eckles and Pottier 2011). Analysing different financial ratios to rank companies for better performance, allows the introduction of different criteria such as profitability, liquidity, solvency, among others.

Therefore, in order to find the best non-life insurance companies from a pool, it is necessary to consider multiple but usually conflicting criteria. Thus, a company may perform well against one criterion and may perform poorly with respect to another criterion. The aggregation of these opposing performances can be achieved by applying multi-criteria methods.

Several studies exist that develop methods for ranking insurance companies (see Ecer and Pamucar 2021 and references therein). These works have applied various methods such as Analytical Hierarchy Process (AHP) (Toloie et al. 2011; Ksenija et al. 2017; Mandić et al. 2017; Beiragh et al. 2020) and Best–Worst Method (Torbati and Sayadi 2018; Akyüz et al. 2020) for determining the importance level of selected criteria. Alternatively, in order to score insurance firms, use has been made of Technique for Order of Preference by Similarity to Ideal Solution (TOPSIS) (Toloie et al. 2011; Abdolvand and Rahpeima 2013; Akhisar and Tunay 2015; Ksenija et al. 2017; Akyüz et al. 2020; Pattnaik et al. 2021), DEA (Sabet and Fadavi 2013; Nourani et al. 2018; Beiragh et al. 2020), Preference Ranking Organization Method for Enrichment of Evaluations (PROMETHEE) (Doumpos et al. 2012; Kazemi and Bardeji 2016), Measurement of Alternatives and Ranking according to Compromise Solution (MARCOS) (Ecer and Pamucar 2021), among others. Although the numerous methods used each have their advantages and disadvantages, it is universally accepted that reliable and robust models are needed to address the ambiguity and vagueness of judgements that arise when assessing insurers.

These studies support the DM in their choice of the best insurance companies and share two main phases: (i) a weighting process to determine the relative importance of the evaluation criteria, and (ii) an aggregation method for obtaining a score representative of the composite performance of each company which allows a ranking of the companies.

The classic AHP is the most used Multi-Criteria Decision Making (MCDM) technique in determining the weights of criteria (Govindan et al. 2014 and Alizadeh and Yousefi 2019) from pairwise comparisons and matrix calculus. Psychology sustains that it is easier and more accurate to express pairwise judgements than opinions simultaneously for all the elements of decision-making. AHP uses a ratio scale, which requires no units for the comparison. The preference scale is composed of the integers 1 to 9 and their reciprocals in order to compare the relative importance of one criterion over another. This linear scale has received criticism and several alternatives have been proposed in the literature (see discussion and references about this issue in Ishizaka and Labib 2011) but according to Saaty (1980, 1990), this scale is the best one for representing weight ratios. Other AHP extensions related to the way pairwise comparisons are expressed have been aimed at offering tools that represent the vagueness, ambiguity and imprecision/uncertainty present in a human's subjective judgments. In this context, interval pairwise comparison judgements have been proposed (Mikhailov 2003, 2004; Chen and Xu 2015; Ren 2018, among others) for overcoming situations where DMs find difficulties in using exact numerical numbers. Once the scale and the mathematical tool representing the pairwise comparisons (crisp numbers, interval values, fuzzy numbers, intuitionist fuzzy numbers, among others) have been chosen, it is necessary to address a key problem of MCDM models: priorities derivation from a pairwise comparison matrix. Different approaches have been proposed based on simple matrix calculus: the method mean of the row, the method of the principal eigenvector (see references in Ishizaka and Labib 2011 and Zhang et al. 2021). In contrast to the aforementioned approaches, most of the prioritisation methods are based on the distance minimisation between the empirical pairwise priority ratio and the corresponding theoretical pairwise priority ratio. Zhang et al. 2021 present a revision of the most famous methods and they propose a novel methodology implying both additive and multiplicative deviation relations. Their approach introduces two norms giving rise to four conic programming models.

In our paper, this first phase of the proposed hybrid MCDM methodology is carried out by an extension of the prioritisation method used in AHP. We work with the linear scale proposed by Saaty (1977) and interval values are used to represent the pairwise comparisons. We extend the Best–Worst (BW) method (Rezaei 2015, 2016; Lahri et al. 2021) for constructing the incomplete interval comparison matrix. This new methodology, introduced as Extended Best–Worst (EBW), is based on a modification of Mikhailov’s approach (2004) for obtaining the priority vector. It consists in solving a minimax problem that gives both the optimal weights and a measure of the consistency between the proposed ratios and the achieved ratios. The second phase, related to the aggregation process, is addressed by applying the Multiple Reference Point (MRP) method which performs two tasks. Firstly, a normalisation of the scores of the companies based on several reference points is carried out, permitting the DMs intervention for the purpose of setting the latter reference points. Secondly, several aggregation procedures based on fully compensatory, non-compensatory and partially compensatory operators allow to obtain a unique global score for the joint performance of each company. These scores share the same interpretation as the scale defined by the set reference points.

The methodology contributions of this study, represented by the Extended Best–Worst Multiple Reference Point (EBW-MRP) method, provides new tools for analysing the global performance of insurance companies. We see the EBW results as a foundation for prioritising criteria in an understanding and easy way for DMs. In addition, the application of a MRP method helps to rank the best alternatives according to expert knowledge.

We apply our methodology to the Spanish non-life insurance market. Since the 1980s, the Spanish insurance market has suffered a restructuring and consolidation process. The consequences of the latter have been studied in papers such as Cummins and Rubio Misas (2006) or Cummins et al (2017) for example. We have chosen the Spanish insurance market, because despite the high impact of the financial crisis in Spain, the non-life insurance sector has managed to grow by more than 6.5% over the period 2007–2017 (Mapfre 2018). This highlights the interest in studying the financial assessment of companies operating in the Spanish non-life insurance market. Between 80 and 83 insurers are considered during the period from 2009 to 2017. The criteria were chosen based on the academic literature and on the data published annually by the Dirección General de Seguros y Fondos de Pensiones (DGSFP).

The rest of the paper is organised as follows. Section 2 presents the proposed methodology, an Extended Best–Worst Multiple Reference Point Method. The next section is devoted to the empirical application. Our database consists of Spanish non-life insurance companies evaluated with respect to eight criteria. The main results of our real application are discussed in Sect. 4, with nine rankings obtained that allow an assessment for the period analysed. Finally, conclusions are discussed in Sect. 5.

## Extended best–worst multiple reference point method

**Fig. 1:**
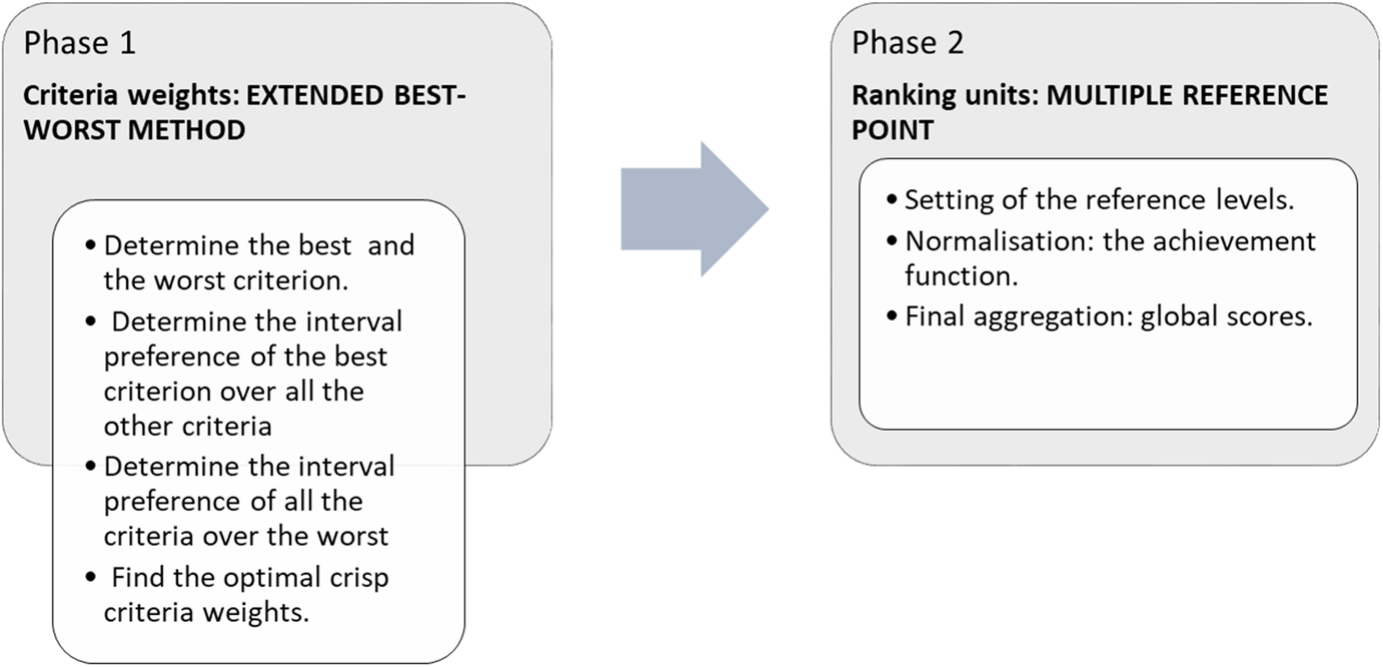
Extended best–worst multiple reference point method

Throughout this section, it will be assumed that we are managing a set of $M$ units $\left\{ {U_{1} ,U_{2} ,...,U_{M} } \right\}$ and $N$ criteria, $\left\{ {C_{1} ,C_{2} ,...,C_{N} } \right\}$, which play an important role in evaluating the units in order to arrive at a decision. To do this, we formulate an Extended Best–Worst Multiple Reference Point (EBW-MRP) method considering the most important criterion (*best*) and the least important criterion (*worst*) for the DM. The procedure is divided into two phases, which are described in detail. In the first phase, a novel BW method is proposed to calculate the criterion weights considering that the relative preference of the *i*-th criterion over the *j*-th criterion is not precisely expressed by the expert since their judgment is imprecise. These weights are incorporated into a MRP model (Ruiz et al. 2020), in the second phase, to obtain the global scores of the units from which a ranking can be obtained (see Fig. 1).

**Phase 1. Extended Best–Worst (EBW) method**

In the classic BW model (see Table 1), it is assumed that the preference value on the *i*-th criterion to the *j*-th criterion determined by experts is accurate. However, in some uncertain/imprecise situations the DM may not be able to provide exact point judgements and she/he expresses her/his preferences through linguistic labels, such as ‘approximately or about $a$’, instead of exact numerical values $a$. The use of fuzzy logic tools (Zadeh 1965; Bellman and Zadeh 1970) may be useful to attempt at mechanisation or formalisation of human reasoning. Some research has been developed to handle vague and uncertain information in the BW method. For example, Mou et al. (2016, 2017) propose an intuitionistic fuzzy multiplicative BW model for multi-criteria group decision making and use the graph theory to find the best and worst criteria; Guo and Zhao (2017) extend the BW method to a fuzzy environment and describe the reference comparisons for the best criterion and for the worst criterion by triangular fuzzy numbers; Aboutorab et al. (2018) address the problem of the uncertainty of real world decisions with Z numbers that integrate in a BW method and obtain triangular fuzzy weights; Pamučar et al (2018) presents a new BW approach where the treatment of uncertainty is based on interval-valued fuzzy-rough numbers; Ren (2018) develop an interval BW method for determining the interval weights of the evaluation criteria and the relative performances of the alternatives with respect to the soft criteria; Ali and Rashid (2019) investigate a BW method in which the uncertain evaluation values are represented by hesitant linguistic term sets; Mi and Liao (2019) implement the weight-determining process by a hesitant fuzzy BW model; Fei et al. (2020) propose an evidential BW model based on the theory of belief functions, which is employed for evaluating hospital service quality.

**Table 1:** Best–worst method (Rezaei 2015)

Step	Description
Step 1	Identify a decision criteria set, $\left\{ {C_{1} ,C_{2} ,...,C_{N} } \right\}$
Step 2	Determine the best and worst decision criteria, $C_{B}$ and $C_{W}$, for the DM
Step 3	Determine the DM’s preference degree of the best criterion over all the other criteria: $A_{B} = \left( {a_{B1} ,a_{B2} ,...,a_{BN} } \right)$
Step 4	Determine the DM’s preference degree of all the criteria over the worst criterion: $A_{W} = \left( {a_{1W} ,a_{2W} ,...,a_{NW} } \right)$
Step 5	Find the optimal solution of criteria weights $\left\{ {w_{1}^{*} ,w_{2}^{*} ,...,w_{N}^{*} } \right\}$:$$\begin{gathered} \min \;\mathop {\max }\limits_{j} \left\{ {\left| {\frac{{w_{B} }}{{w_{j} }} - a_{Bj} } \right|,\left| {\frac{{w_{j} }}{{w_{W} }} - a_{jW} } \right|} \right\} \hfill \\ s.t.\;\;\sum\limits_{j = 1}^{n} {w_{j} = 1,\;\;w_{j} > 0,\;{\text{for all }}j} \hfill \\ \end{gathered}$$

In this paper, the uncertain/imprecise judgements are represented by interval ratios (Mikhailov 2003, 2004; Arbel and Vargas 2007; Wang and Elhag 2007; Yue, 2012; Ahn 2017; Ren 2018; Acuña-Soto et al. 2019). A novel weighting problem is then formulated and solved as a max–min mathematical programming problem for obtaining an optimal crisp weighting vector that maximises the overall degree of satisfaction of the DM.

To derive priorities from uncertain judgements, Saaty and Vargas (1987) construct an interval reciprocal comparison matrix of the type:

1 $$A = \left( {\begin{array}{*{20}c} 1 & {a_{12} } & \cdots & {a_{1N} } \\ {a_{21} } & 1 & \cdots & {a_{2N} } \\ \vdots & \vdots & \ddots & \vdots \\ {a_{N1} } & {a_{N2} } & \cdots & 1 \\ \end{array} } \right)$$

where $a_{ij} = \left[ {l_{ij}^{{}} ,u_{ij}^{{}} } \right]$ represents the relative preference of criterion $i$ to criterion $j$, which is an interval judgement.$l_{ij}^{{}}$ and $u_{ij}^{{}}$ are the lower and the upper bounds of the interval. The range of bounds is assumed to be between $1/9$ and $9$ inclusive (Saaty 1977), taking into account that $a_{ij} = a_{ji}^{ - 1}$ and the operations on closed intervals we have:

2 $$a_{ij} = \left[ {l_{ij}^{{}} ,u_{ij}^{{}} } \right] = \left[ {\frac{1}{{u_{ji} }},\frac{1}{{l_{ji} }}} \right]$$

### **Definition 1**

An interval pairwise comparison $a_{ij}$ is defined as a *reference comparison* if $i$ is the best criterion and/or $j$ is the worst criterion.

According to Rezaei (2015), $N(N - 1)/2$ interval pairwise comparisons are not needed to obtain the complete interval comparison matrix and it is sufficient to determine the $2N - 3$ reference comparisons. This is the basis principle of the interval weighting method that we are going to formulate below.

The steps of the EBW method that can be used to obtain the weights of the $N$ criteria, $\left( {w_{1}^{*} ,w_{2}^{*} ,...,w_{N}^{*} } \right)$, are described in what follows:

*Step 1.* Determine the best criterion, $C_{B}$, and the worst criterion, $C_{W}$.

*Step 2.* Determine the preference of the best criterion over all the other criteria by mean of interval judgements. The resulting best-to-others interval-vector would be:

3 $$A_{B} = \left( {a_{B1} ,a_{B2} ,...,a_{BN} } \right) = \left( {\left[ {l_{B1}^{{}} ,u_{B1}^{{}} } \right],\left[ {l_{B2}^{{}} ,u_{B2}^{{}} } \right],...,\left[ {l_{BN}^{{}} ,u_{BN}^{{}} } \right]} \right)$$

where $a_{Bj} = \left[ {l_{Bj}^{{}} ,u_{Bj}^{{}} } \right]$ indicates the interval preference of the best criterion $C_{B}$ over the criterion $j$.

*Step 3. Determine* the preference of all the criteria over the worst criterion by mean of interval judgements. The resulting others-to-worst interval-vector would be:

4 $$A_{W} = \left( {a_{1W} ,a_{2W} ,...,a_{NW} } \right) = \left( {\left[ {l_{1W}^{{}} ,u_{1W}^{{}} } \right],\left[ {l_{2W}^{{}} ,u_{2W}^{{}} } \right],...,\left[ {l_{NW}^{{}} ,u_{NW}^{{}} } \right]} \right)$$

where $\left[ {l_{jW}^{{}} ,u_{jW}^{{}} } \right]$ indicates the interval preference of the criterion $j$ over the worst criterion $C_{W}$.

*Step 4.* Find the optimal crisp weights vector $w^{*} = \left( {w_{1}^{*} ,w_{2}^{*} ,...,w_{N}^{*} } \right)$.

In this work, a crisp priority vector $w = \left( {w_{1}^{{}} ,w_{2}^{{}} ,...,w_{N}^{{}} } \right)$ is admissible with respect to the best-to-others interval-vector, $A_{B}$, and the others-to-worst interval-vector, $A_{W}$, if it verifies

5 $$l_{Bj} \tilde{ \le }\frac{{w_{B}^{{}} }}{{w_{j}^{{}} }}\tilde{ \le }u_{Bj} ,\quad j = 1,...,N,j \ne B$$

6 $$l_{jW} \tilde{ \le }\frac{{w_{j}^{{}} }}{{w_{W}^{{}} }}\tilde{ \le }u_{jW} ,\quad j = 1,...,N,j \ne W$$

where $\tilde{ \le }$ denotes the fuzzified version of $\le$ and reads ‘approximately less than or equal to’.

The weights $w_{1}^{*} ,w_{2}^{*} ,...,w_{N}^{*}$ will be *optimal* if they satisfy the fuzzy inequalities (5) and (6) with the highest degree of membership. Therefore, in order to find the optimal weights a fuzzy nonlinear problem must be solved.

In order to linearise the fuzzy problem, (5) and (6) are transformed into the following fuzzy linear inequalities:

7 $$- w_{B} + l_{Bj} w_{j} \tilde{ \le }0{\text{ and }}\;w_{B} - u_{Bj} w_{j} \tilde{ \le }0,\quad j = 1,...,N,j \ne B$$

8 $$- w_{j} + l_{jW} w_{W} \tilde{ \le }0{\text{ and }}\;w_{j} - u_{jW} w_{W} \tilde{ \le }0,\quad j = 1,...,N,j \ne W$$

The range of approximate satisfaction of (7) and (8) can be defined as extended intervals $\left[ {0,d_{Bj}^{l} } \right]$, $\left[ {0,d_{Bj}^{u} } \right]$, $\left[ {0,d_{jW}^{l} } \right]$ and $\left[ {0,d_{jW}^{u} } \right]$ where $d_{Bj}^{l}$, $d_{Bj}^{u}$, $d_{jW}^{l}$ and $d_{jW}^{u}$ are tolerance parameters for the corresponding intervals.^1^

**Fig. 2:**
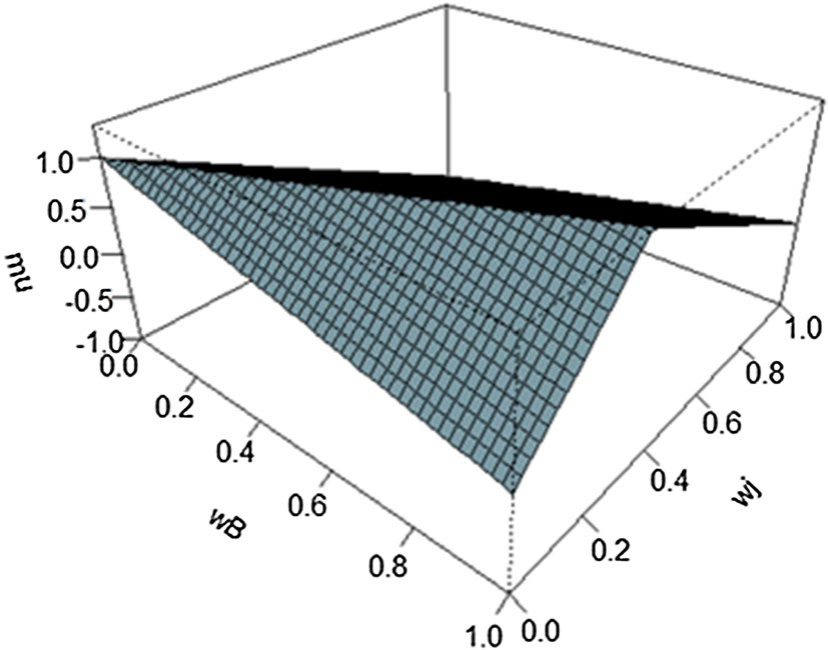
Linear satisfaction function

As the two constraints in (7) relate to the same interval, we can represent them as a linear satisfaction function (see Fig. 2) corresponding to the lower and upper bounds that expresses the DM’s satisfaction with its accomplishment (Mikhailov 2004; Chen and Xu 2015):

9 $$\mu_{Bj} (w) = \left\{ {\begin{array}{*{20}c} {1 - \frac{{( - w_{B} + l_{Bj} w_{j} )}}{{d_{Bj}^{l} }} = 1 + \frac{{\frac{{w_{B} }}{{w_{j} }} - l_{Bj} }}{{\frac{{d_{Bj}^{l} }}{{w_{j} }}}},} & {\frac{{w_{B} }}{{w_{j} }} \le m_{Bj} } \\ {1 - \frac{{(w_{B} - u_{Bj} w_{j} )}}{{d_{Bj}^{u} }} = 1 + \frac{{u_{Bj} - \frac{{w_{B} }}{{w_{j} }}}}{{\frac{{d_{Bj}^{u} }}{{w_{j} }}}},} & {\frac{{w_{B} }}{{w_{j} }} \ge m_{Bj} } \\ \end{array} } \right.$$

where $m_{Bj} = \frac{{\frac{1}{{d_{Bj}^{l} }}l_{Bj} + \frac{1}{{d_{Bj}^{u} }}u_{Bj} }}{{\frac{1}{{d_{Bj}^{l} }} + \frac{1}{{d_{Bj}^{u} }}}}$ is the extended middle of the interval $\left[ {l_{Bj}^{{}} ,u_{Bj}^{{}} } \right]$ where the greatest satisfaction is achieved.

This function has a maximum, $\mu_{Bj}^{\max }$, when ${{w_{B} } \mathord{\left/ {\vphantom {{w_{B} } {w_{j} }}} \right. \kern-\nulldelimiterspace} {w_{j} }} = m_{Bj}$. In this case the DM should be ‘*most satisfied*’. Otherwise, the DM is ‘*satisfied*’ when $l_{Bj} < {{w_{B} } \mathord{\left/ {\vphantom {{w_{B} } {w_{j} < u_{Bj} }}} \right. \kern-\nulldelimiterspace} {w_{j} < u_{Bj} }}$, and she/he is ‘*partially satisfied*’ when ${{w_{B} } \mathord{\left/ {\vphantom {{w_{B} } {w_{j} }}} \right. \kern-\nulldelimiterspace} {w_{j} }}$ takes a value within the admissible interval $\left[ {l_{Bj} - {{d_{Bj}^{l} } \mathord{\left/ {\vphantom {{d_{Bj}^{l} } {w_{j} }}} \right. \kern-\nulldelimiterspace} {w_{j} }},u_{Bj} + {{d_{Bj}^{u} } \mathord{\left/ {\vphantom {{d_{Bj}^{u} } {w_{j} }}} \right. \kern-\nulldelimiterspace} {w_{j} }}} \right]$. Finally, ${{w_{B} } \mathord{\left/ {\vphantom {{w_{B} } {w_{j} }}} \right. \kern-\nulldelimiterspace} {w_{j} }} \notin \left[ {l_{Bj} - {{d_{Bj}^{l} } \mathord{\left/ {\vphantom {{d_{Bj}^{l} } {w_{j} }}} \right. \kern-\nulldelimiterspace} {w_{j} }},u_{Bj} + {{d_{Bj}^{u} } \mathord{\left/ {\vphantom {{d_{Bj}^{u} } {w_{j} }}} \right. \kern-\nulldelimiterspace} {w_{j} }}} \right]$ indicates ‘*dissatisfaction*’ with the pair $(w_{B} ,w_{j} )$.

We have a similar function for the two constraints in (8):

10 $$\mu_{jW} (w) = \left\{ {\begin{array}{*{20}c} {1 - \frac{{( - w_{j} + l_{jW} w_{W} )}}{{d_{jW}^{l} }} = 1 + \frac{{\frac{{w_{j} }}{{w_{W} }} - l_{jW} }}{{\frac{{d_{jW}^{l} }}{{w_{W} }}}},} & {\frac{{w_{j} }}{{w_{W} }} \le m_{jW} } \\ {1 - \frac{{(w_{j} - u_{jW} w_{W} )}}{{d_{jW}^{u} }} = 1 + \frac{{u_{jW} - \frac{{w_{j} }}{{w_{W} }}}}{{\frac{{d_{jW}^{u} }}{{w_{W} }}}},} & {\frac{{w_{j} }}{{w_{W} }} \ge m_{jW} } \\ \end{array} } \right.$$

The function (10) has a maximum, $\mu_{jW}^{\max }$, when ${{w_{j} } \mathord{\left/ {\vphantom {{w_{j} } {w_{W} }}} \right. \kern-\nulldelimiterspace} {w_{W} }} = m_{jW}$.^2^ In this case the DM should be ‘*most satisfied*’. Otherwise, the DM is ‘*satisfied*’ when $l_{jW} < {{w_{j} } \mathord{\left/ {\vphantom {{w_{j} } {w_{W} < u_{jW} }}} \right. \kern-\nulldelimiterspace} {w_{W} < u_{jW} }}$, and she/he is ‘*partially satisfied*’ when ${{w_{j} } \mathord{\left/ {\vphantom {{w_{j} } {w_{W} }}} \right. \kern-\nulldelimiterspace} {w_{W} }}$ takes a value within the admissible interval $\left[ {l_{jW} - {{d_{jW}^{l} } \mathord{\left/ {\vphantom {{d_{jW}^{l} } {w_{W} }}} \right. \kern-\nulldelimiterspace} {w_{W} }},u_{jW} + {{d_{jW}^{u} } \mathord{\left/ {\vphantom {{d_{jW}^{u} } {w_{W} }}} \right. \kern-\nulldelimiterspace} {w_{W} }}} \right]$. Finally, ${{w_{j} } \mathord{\left/ {\vphantom {{w_{j} } {w_{W} }}} \right. \kern-\nulldelimiterspace} {w_{W} }} \notin \left[ {l_{jW} - {{d_{jW}^{l} } \mathord{\left/ {\vphantom {{d_{jW}^{l} } {w_{W} }}} \right. \kern-\nulldelimiterspace} {w_{W} }},u_{jW} + {{d_{jW}^{u} } \mathord{\left/ {\vphantom {{d_{jW}^{u} } {w_{W} }}} \right. \kern-\nulldelimiterspace} {w_{W} }}} \right]$ indicates ‘*dissatisfaction*’ with the pair $(w_{j} ,w_{W} )$.

Taking into account the above satisfaction functions, we can solve the weighting problem applying the Zimmermann fuzzy programming approach (1976). To do this, the feasible set of weighting vectors is defined:

11 $$Q^{n - 1} = \left\{ {\left( {w_{1}^{{}} ,w_{2}^{{}} ,...,w_{N}^{{}} } \right){{} \mathord{\left/ {\vphantom {{} {}}} \right. \kern-\nulldelimiterspace} {}}w_{j} > 0,\;\sum\limits_{j = 1}^{N} {w_{j} = 1} } \right\}$$

The fuzzy subset $\tilde{P}$, whose membership function $\mu_{{\tilde{P}}} (w)$ is the intersection of the satisfaction functions defined in (9) and (10):

12 $$\mu_{{\tilde{P}}} (w) = \mathop {\min }\limits_{j} \left\{ {\mu_{Bj} (w),\mu_{jW} (w)} \right\}$$

This membership function represents the *overall satisfaction* of the DM with a specific weighting vector $w$.

**Definition 2**

A vector $w^{*} = \left( {w_{1}^{*} ,w_{2}^{*} ,...,w_{N}^{*} } \right)$ is an *optimal weighting vector* with respect to the best-to-others interval-vector, $A_{B}$, and the others-to-worst interval-vector, $A_{W}$, if it maximises the *overall* degree of satisfaction $\mu_{{\tilde{P}}} (w)$. i.e.,

13 $$\mu_{P} (w^{*} ) = \mathop {\max }\limits_{{w \in Q^{n - 1} }} \mu_{P} (w) = \mathop {\max }\limits_{{w \in Q^{n - 1} }} \mathop {\min }\limits_{j} \left\{ {\mu_{Bj} (w),\mu_{jW} (w)} \right\}$$

As is well known, this problem is equivalent to solving the following linear program:

14 $$\begin{gathered} \max \, \lambda \hfill \\ subject \, to \hfill \\ d_{Bj}^{l} \,\lambda - w_{B} + l_{Bj} w_{j} \le d_{Bj}^{l} ,\quad j = 1,2,...,N,j \ne B \hfill \\ d_{Bj}^{u} \,\lambda + w_{B} - u_{Bj} w_{j} \le d_{Bj}^{u} ,\quad j = 1,2,...,N,j \ne B \hfill \\ d_{jW}^{l} \,\lambda - w_{j} + l_{jW} w_{W} \le d_{jW}^{l} ,\quad j = 1,2,...,N,j \ne W \hfill \\ d_{jW}^{u} \,\lambda + w_{j} - u_{jW} w_{W} \le d_{jW}^{u} ,\quad j = 1,2,...,N,j \ne W \hfill \\ \sum\limits_{j = 1}^{N} {w_{j} = 1,\quad w_{j} > 0,\quad \forall j} \hfill \\ \end{gathered}$$

The optimal solution of (14) is a vector $\left( {w^{*} ,\lambda^{*} } \right)$ whose first component represents the weighting vector that maximises the degree of membership of the aggregated function $\mu_{{\tilde{P}}} (w)$, whereas $\lambda^{*}$ measures the degree of overall satisfaction of the DM with the optimal solution $w^{*}$.

The optimal value $\lambda^{*}$ is also an indicator for measuring the consistency of the DM judgements and, for a given set of interval judgements, depends on the values of the tolerance parameters.

### **Definition 3**

The DM interval judgements are *strongly inconsistent* if the optimal value $\lambda^{*}$ of (14) is negative. In this case the solution ratios are outside the extended intervals.

The following proposition avoids the problems of strong inconsistency in the DM interval judgments.

### **Proposition 1**

(Mikhailov 2004). The solution of (14) verifies $\lambda^{*} \ge 0$, if all deviation parameters $d_{Bj}^{l} ,d_{Bj}^{u} ,d_{jW}^{l} ,d_{jW}^{u}$ is greater than or equal to $d^{*}$, where $d^{*}$ is the solution of the following linear problem:

15 $$\begin{gathered} \min \, d \hfill \\ subject \, to \hfill \\ - w_{B} + l_{Bj} w_{j} \le d,\quad j = 1,2,...,N,j \ne B \hfill \\ w_{B} - u_{Bj} w_{j} \le d,\quad j = 1,2,...,N,j \ne B \hfill \\ - w_{j} + l_{jW} w_{W} \le d,\quad j = 1,2,...,N,j \ne W \hfill \\ w_{j} - u_{jW} w_{W} \le d,\quad j = 1,2,...,N,j \ne W \hfill \\ \sum\limits_{j = 1}^{N} {w_{j} = 1,\quad w_{j} > 0,\quad \forall j} ,\quad d > 0 \hfill \\ \end{gathered}$$

The weights obtained in this *Phase 1* are incorporated into a Multiple Reference Point model (Ruiz et al. 2020; García-Bernabeu et al., 2021; Cabello et al. 2021; Boggia et al. 2022) for ranking and choosing the best unit.

**Phase 2. Multiple Reference Point to calculate the global scores of the units ** (Ruiz et al., 2020).

Let us denote by $x_{ij}$ the value of the $i$-th unit for the criterion $j$-th.

*Step 1.* Setting of the reference levels.

Let us denote by $q_{j}^{0}$ and $q_{j}^{n + 1}$, respectively, the minimum and maximum values that criterion $j$ can take. For each criterion $j$, the DM gives $n$ reference levels, $q_{j}^{1} ,q_{j}^{2} ,...,q_{j}^{n} ,$ which define the performance levels of criterion $j$ (e.g., very low, low, medium, high, very high or very poor, poor, fairly good, very good). Wierzbicki et al. (2000) mention several ways for establishing these reference levels. They can be defined in an absolute way by experts, in a relative way, applying a statistical scheme, or by setting all the reference levels equal to certain percentages of their respective criteria ranges.

Therefore, the $(n + 2)$-dimensional vector

16 $${\varvec{q}}_{{\varvec{j}}} = \left( {q_{j}^{0} ,q_{j}^{1} ,q_{j}^{2} ,...,q_{j}^{n} ,q_{j}^{n + 1} } \right)$$

contains all the information relative to the reference levels of the criterion $j$. These reference levels can naturally define performance levels for each criterion, in absolute or relative terms, and the corresponding distance function measures the position of each unit with respect to these levels.

*Step 2.* Normalisation: the achievement function.

We assume a set of $n + 2$ real values $\alpha_{{}}^{0} ,\alpha_{{}}^{1} ,...,\alpha_{{}}^{n} ,\alpha_{{}}^{n + 1}$ which define a common measurement scale for all the criteria. A piece-wise linear achievement function is used to turn each criterion $j$ to the scale defined by the values $\alpha^{k}$.

If the *j* criterion is of type “the more, the better” we consider:

17 $$\begin{gathered} s_{j} \left( {x_{ij} ,{\varvec{q}}_{{\varvec{j}}} } \right) = \alpha^{k - 1} + \frac{{\alpha^{k} - \alpha^{k - 1} }}{{q_{j}^{k} - q_{j}^{k - 1} }}\left( {x_{ij} - q_{j}^{k - 1} } \right)\quad {\text{if}}\quad x_{ij} \in \left[ {q_{j}^{k - 1} ,q_{j}^{k} } \right],\quad \hfill \\ \, k = 1,2,...,n + 1 \hfill \\ \end{gathered}$$

In this case $s_{j} \left( {x_{ij} ,{\varvec{q}}_{{\varvec{j}}} } \right)$ is a an increasing piece-wise linear function. Therefore, the achievement function $s_{j}$ of criterion $j$ takes values between $\alpha^{k - 1}$ and $\alpha^{k}$ if the unit achieves values between $q_{j}^{k - 1}$ and $q_{j}^{k}$ for the criterion $j$.

If the *j* criterion is of type “the lower, the better”

18 $$s_{j} \left( {x_{ij} ,q_{j} } \right) = \alpha^{n + 2 - k} - \frac{{\alpha^{n + 2 - k} - \alpha^{n + 1 - k} }}{{q_{j}^{k} - q_{j}^{k - 1} }}\left( {x_{ij} - q_{j}^{k - 1} } \right)\;if\;x_{ij} \in \left[ {q_{j}^{k - 1} ,q_{j}^{k} } \right],\;k = 1,2,...,n + 1$$

In this case $s_{j} \left( {x_{ij} ,{\varvec{q}}_{{\varvec{j}}} } \right)$ is a decreasing piece-wise linear function. Therefore, the achievement function $s_{j}$ of criterion $j$ takes values between $\alpha^{n + 1 - k}$ and $\alpha^{n + 2 - k}$ if the unit achieves values between $q_{j}^{k - 1}$ and $q_{j}^{k}$ for the criterion $j$.

*Step 3.* Final aggregation: global scores.

Considering the weights obtained in Phase 1, $w^{*} = \left( {w_{1}^{*} ,w_{2}^{*} ,...,w_{N}^{*} } \right)$, and the achievement functions (17) and (18), we can build the global score of each unit. At this stage, we can obtain different types of composite measures.

The weak composite measure of unit $i,$ $WS_{i} ,$ allowing for full compensation among the criteria, is obtained using an additive weighted aggregation:

19 $$WS_{i} = \sum\nolimits_{j = 1}^{N} {w_{j}^{*} } s_{j} \left( {x_{ij} ,{\varvec{q}}_{{\varvec{j}}} } \right)$$

as $\sum\nolimits_{j = 1}^{N} {w_{j}^{*} = 1}$ then $WS_{i} \in \left[ {\alpha^{0} ,\alpha^{n + 1} } \right]$ which can be easily interpreted as the composite performance of the unit $i$ with respect to the reference levels ${\varvec{q}}_{{\varvec{j}}}$.

The strong composite measure of unit $i,$ $SS_{i} ,$ does not allow any compensation. In this case, following Ruiz et al. (2020), it is possible to modify the achievement functions (17) and (18) so that poor performance on a given criterion is not so bad if the criterion is not very important to the DM. For this, a normalisation of the weights is used, so that the highest weight takes the value 1:

20 $$\varpi_{j}^{*} = \frac{{w_{j}^{*} }}{{\mathop {\max }\nolimits_{k = 1,...,N} \left\{ {w_{k}^{*} } \right\}}}$$

Then, the modified achievement function takes the form:

21 $$\begin{gathered} \overline{s}_{j} \left( {x_{ij} ,{\varvec{q}}_{{\varvec{j}}} } \right) = \alpha^{k} + \left( {s_{j} \left( {x_{ij} ,{\varvec{q}}_{{\varvec{j}}} } \right) - \alpha^{k} } \right)\varpi_{j}^{*} \quad {\text{if}}\quad s_{j} \left( {x_{ij} ,{\varvec{q}}_{{\varvec{j}}} } \right) \in \left[ {\alpha_{{}}^{k - 1} ,\alpha_{{}}^{k} } \right],\quad \hfill \\ \, k = 1,2,...,n + 1 \hfill \\ \end{gathered}$$

Making use of (21) it is possible to build the strong composite measure as:

22 $$SS_{i} = \mathop {\min }\limits_{j = 1,...,N} \left\{ {\overline{s}_{j} \left( {x_{ij} ,{\varvec{q}}_{{\varvec{j}}} } \right)} \right\}$$

Finally, we propose building new composite measures of unit $i,$ $PS_{i} ,$ for different compensation degrees considering the following convex linear combination of the weak and strong composite measure:

23 $$PS_{i} = \lambda_{i} \;WS_{i} + \left( {1 - \lambda_{i} } \right)SS_{i}$$

The coefficient $\lambda_{i}$ associated to unit $i$ is obtained as follows:

*Step 1:* Set a threshold $0 < th \le 1$.

*Step 2*: Rank (decreasing order) the criteria according to their weights: $C_{p(1)} ,C_{p(2)} ,...,C_{p(N)}$, where *p* is a permutation of $1,2,...,N$.

*Step 3:* Identify the minimum set of “important” criteria, $C_{p(1)} ,C_{p(2)} ,...,C_{p(H)} ,$ such that the sum of their weights is higher or equal to the threshold:

$$\sum\limits_{j = 1}^{H} {w_{p(j)}^{*} } \ge th$$

*Step 4:* Calculate the coefficient $\lambda_{i}$ as below:

$$\lambda_{i} = \frac{{\sum\nolimits_{j = 1}^{H} {s_{j} \left( {x_{ij} ,{\varvec{q}}_{{\varvec{j}}} } \right)} }}{{H\;\alpha^{n + 1} }}$$

Indeed, $0 \le \lambda_{i} \le 1$. Note that $\lambda_{i}$ is close to 1 when the unit *i* achieves good results for the most important criteria, therefore the weighted mean $WS_{i}^{{}}$ is a suitable composite measure of performance. On the contrary, $\lambda_{i}$ is close to 0 when the unit *i* achieves bad results for the most important criteria and therefore, the compensation of the weighted mean should be corrected with a higher coefficient for the strong measure $SS_{i}^{{}}$.

We observe $PS_{i}^{{}} = \alpha^{n + 1}$ if and only if the unit *i* achieves the score $\alpha^{n + 1}$ for all criteria and $PS_{i}^{{}} = \alpha^{0}$ if and only if the unit *i* achieves the score $\alpha^{0}$ for all criteria.

The value of the global score obtained with the above composite measures is influenced by the choice of the reference levels ${\varvec{q}}_{{\varvec{j}}}$.

The set of units can now be ranked according to the descending order of the global scores obtained by (19), (22) or (23).

In the next section we apply the proposed Extended Best–Worst Multiple Reference Point (EBW-MRP) method for ranking non-life insurance companies operating in Spain.

## Case study: spanish non-life insurance companies

In 2017, the Spanish insurance market with a total volume of total direct premiums amounting to €62,451 million ($70,547 million) ranked 15th worldwide in front of other European countries such as Switzerland, Sweden, Belgium, Finland or Portugal, among others 3. Despite this, in line with other international insurance markets, the recent crisis had an important impact on the Spanish insurance market. Nevertheless, the Spanish sector has proved robust, managing to overcome this economic cycle in a solid way (Manzano 2017). This is reflected by the evolution of the different financial ratios that it has presented in recent years. Specifically, the Non-Life insurance market, has seen an accumulated growth of 6.8% over the period 2007–2017, while in parallel Spanish GDP grew by 7.7% (Mapfre 2018). This economic growth is linked to an increase in the consumption capacity of homes and companies, therefore contributing towards the growth of the Non-Life insurance business.

### Database

The present research studies the performance of Non-Life Spanish insurers over the period 2009–2017 (see Table A1 in the Appendix). The data have been collected from the Balance Sheets and Accounts of insurance entities that the Spanish regulatory and supervisory authority, the Dirección General de Seguros y Fondos de Pensiones (DGSFP), publishes annually.

#### Non-life insurance companies

In Spain, insurance companies can operate in life, non-life or simultaneously in both life and non-life branches of insurance. In this paper, we focus on those firms that operate exclusively in non-life business between the years 2009–2017. This criterion has the advantage that it makes the insurers more comparable. But, at the same time, it has the disadvantage that insurers such as Mapfre, Mutua Madrileña, or Allianz among others, with important market shares in Spain, but operating simultaneously in the life and non-life business, can be omitted. Table 2 shows the number of firms considered each year. In 2009, 80 firms operate exclusively in non-life business in the Spanish market. In 2011, Segurcaixa is incorporated to this group because the firm split the business of life and non-life into two different insurers. The same happened with Sabadell in 2014 and Asisa in 2015.

**Table 2:** Non-life insurance companies for the period 2009–2017

	2009	2010	2011	2012	2013	2014	2015	2016	2017
N. Firms	80	80	81	81	81	82	83	83	83
Added firms			SEGIRCAIXA			SABADELL	ASISA		

In Spain, insurers can have two organizational forms: private (stocks) and mutuals. The literature has always considered this a determining aspect of insurers’ performance, with stocks being more profitable than mutuals, and with better performance (Cummins and Nini 2002; Gaganis et al. 2015). Given that among the companies analysed in our study, both types coexist (23 mutuals appear in our database for all years), we will also analyse whether or not our performance ranking supports this hypothesis.

#### Criteria

Similar to other research in the field (see Table 3), in order to approach the performance and profitability of insurers, different financial ratios have been calculated, all of which are typically used by international organisms such as The International Association of Insurance Supervisors or the OCDE to evaluate the global insurance market.

**Table 3:** Formula and references for the criteria

Criteria formula	References
${\text{Premium Growth}}\;_{t} = \frac{{{\text{Total Net Premium}}_{t} - {\text{Total Net Premium}}_{t - 1} }}{{{\text{Total Net Premium}}_{t - 1} }}$	Eling and Jia (2019); Saeed and Khurram (2015)
${\text{Loss Ratio}}_{{\text{t}}} { = }\frac{{{\text{Total Net Claims}}_{{\text{t}}} }}{{{\text{Total Net Premium}}_{{\text{t}}} }}$	Eling and Jia (2019); Akuffo et al. (2016); Venkateswarlu and Bhishma (2016); Dar and Thaku (2015)
${\text{Expenses Ratio}}_{t} { = }\frac{{{\text{Total Underwritting Expenses}}_{t} }}{{{\text{Total Net Premium}}_{t} }}$	Venkateswarlu and Bhishma (2016); Dar and Thaku (2015)
${\text{Combined Ratio}}_{t} {\text{ = Loss Ratio}}_{{\text{t}}} + {\text{Expenses Ratio}}_{t} \,$	Eling and Jia (2019); Venkateswarlu Bhishma (2016); Dar and Thaku (2015)
${\text{ROE}}_{t} = \frac{{{\text{Profit before taxes}}_{t} }}{{{\text{Equity}}_{t} }}$	Eling and Jia (2019); Bilbao et al. (2019); Cummins et al. (2017); Dar and Thaku (2015)
${\text{Technical Ratio}}_{t} = \frac{{{\text{Profit Non - life Activity}}_{t} }}{{{\text{Net Premiums Writting}}_{t} }}$	Kaya (2016); Akhisar and Tunay (2015); Kung et al (2006); Diacon et al. (2002)
${\text{No}}{\text{. of Business lines}} = n_{t} \in \left\{ {1,2,3,...,18} \right\}$	Cummins and Xie (2013); Cummins et al. (2010)

*Premium growth ratio*: it reflects the evolution of the yearly premium. If for an insurer company this ratio is above the mean of the market, it means that this insurer is expanding its share market.

*Loss ratio*: it indicates the percentage of premiums used to pay claims. The smaller the better. A high loss ratio would indicate poor risk management by the insurer and the need for greater control over future payments as well as the process of underwriting policies.

*Expenses ratio*: it reflects that part of the premium employed to pay the underwriting expenses, including acquisitions cost, commissions, administrative and general management expenses. This ratio reflects an insurer's ability to manage its daily activity. The lower this ratio is, the more efficient is the insurer is in its management, this enabling it to obtain greater benefits.

*Combined ratio*: it is the sum of the loss and expenses ratios. This ratio shows a first approximation to the technical profitability of the insurer. Lower values of this ratio would imply that the entity chooses its policyholders well and manages expenses better. If the ratio is greater than 1, it implies that the expenses are greater than the premiums, which means that the insurer is not obtaining benefits with its underwriting activity; either because it has many claims or because expenses are too high. Insurers should compensate these losses with income derived from financial investments, which in non-life insurance have a less relevant role than in life insurance.

*Return on equity* (*ROE*): This ratio explains the relation of profit to shareholder capital. It is one of the ratios commonly used in the financial analysis of any firm. High values of this ratio indicate that the company is able to generate a lot of profit with less capital.

*Technical ratio*: This measures how much profit the insurer makes in relation to the premiums. It indicates the performance of the insurer derived solely from its underwriting activity. The higher the ratio, the greater its financial strength, as the company can generate more profit per income received, thus indicating good management and business efficiency.

*Number of Insurance Business Lines*: among Non-Life Insurance, up to 19 different lines of business can be distinguished: health, dependency, automobile, home … This variable includes the number of business lines in which each observed insurer operates. It gives us a proxy of the diversification grade of the insurer.

Table 4 shows the main statistics of the criteria calculated for the Spanish Non-Life Insurance market for the period 2009–2017.^4^

**Table 4:** Statistics of the criteria

	Premium growth	Share market	Loss ratio	Expenses ratio	Combined ratio	ROE	Technical ratio	No. of Business lines
Min	− 0.41102	0	0.11127	0.02177	0.47934	− 0.64620	− 0.45142	1
1st Quartile	− 0.03054		0.55382	0.13051	0.84196	0.02904	0.03326	2
Mean	0.04903		0.68052	0.24689	0.92741	0.10677	0.10053	5.22
Median	0.01110		0.71683	0.21251	0.93336	0.09244	0.08294	3.83
3º Quartile	0.05063		0.81953	0.32731	0.99292	0.17490	0.16035	8.19
Max	1.93293	1	1.23166	0.71177	1.48811	0.59961	0.61183	18.89
Stand. Dev	0.27841		0.21768	0.15646	0.15869	0.16624	0.14739	4.28

**Fig. 3:**
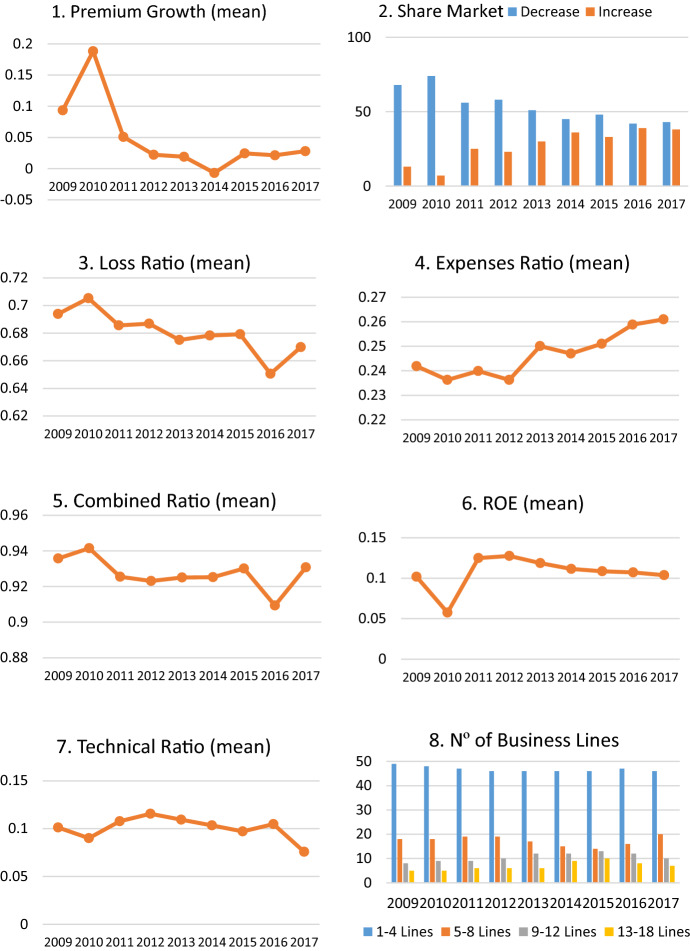
Evolution of the different criteria from 2009 to 2017

Figure 3 shows the evolution of the different criteria throughout the analysed period. An important decrease in premium growth is observed between 2010 and 2013, coinciding with the economic crisis lived by Spain. In 2014 there was a change in the trend of premium growth which continued in 2017, as stated in the Mapfre (2018) report. The evolution of the variations in market shares confirms this trend given that, in recent years, many companies have improved their market share, probably due to the increase in the premiums written linked to the upturn of the Spanish economy.

The analysis of the Combined Ratio shows an efficient and healthy non-life insurance sector, as its mean values remained below 1 over the whole period. The big drop of this ratio in 2016 is explained by an important decrease in claims for this year also seen in the loss ratio. At the same time, the expenses ratio has seen an increase since 2004, parallel to the increase in premium writing in the market.

The profitability of non-life insurers can be approximated through their Technical Ratio and ROE. Both measures indicate that Spanish non-life insurers are good managers, generating an average profitability of 10% throughout the period, both on equity (ROE) and in terms of technical activity (Technical Ratio). In 2010, the effects of the economic crisis were felt in the Spanish insurance market and a significant drop in both the ROE and technical ratio occurred. Despite this, profitability levels recovered from 2011 onwards, once again demonstrating the financial strength of the sector.

Figure 3 (graphic 8) shows the number of business lines in which non-life insurers operate. This variable is stable along the period, with almost 60% of companies only working in up to 3 branches. This is probably due to a large number of companies being specialised in specific branches such as: health, credit and surety or legal defence, among others.

### Extended Best–Worst multiple reference point method for the non-life insurance companies

#### Extended Best–Worst (EBW) Method to calculate the criteria weights.

In order to determine the weights of each criterion, model (14) will be solved. To do this, preferential parameters should be set. The ranking of the importance of the decision criteria is established according to expert opinion and is shown in the first column of Table 5 in descending order, the best criterion is the ROE and the worst is the number of business lines. Reference interval preferential ratios used for solving (14) are displayed in Table 5.

**Table 5:** Pairwise preferential ratios

Criteria	$l_{Bj}$	$u_{Bj}$	$l_{jW}$	$u_{jW}$
ROE	1	1	7	8
Technical ratio	1	2	6	7
Combined ratio	2	3	5	6
Expense ratio	3	4	4	5
Loss ratio	4	5	3	4
Share market	5	6	2	3
Premium growth	6	7	1	2
No. of business lines	7	8	1	1

The weights obtained by (14) using the deviation parameter^5^$d = 0.1$ are displayed in the last column of Table 6, the optimum value, $\lambda^{*} ,$ is equal to 0.75 showing that the obtained ratios are within the extended intervals but not in the strict intervals. The value used for the deviation parameter *d* assures a degree of overall satisfaction $\lambda$ greater than zero because the solution for model (15) gives a lower bound for the parameter *d* equal to 0.0254. In order to visualise the results of the EBW method in our application, Table 6 shows the preferential intervals established and the achieved ratios as well as the left and right spreads corresponding to these intervals.

**Table 6:** Solutions of the model (14)

Criteria	$l_{Bj}$	$u_{Bj}$	Achieved ratio ${{w_{B} } \mathord{\left/ {\vphantom {{w_{B} } {w_{j} }}} \right. \kern-\nulldelimiterspace} {w_{j} }}$	${d \mathord{\left/ {\vphantom {d {w_{j} }}} \right. \kern-\nulldelimiterspace} {w_{j} }}$	Weights
ROE	1	1	1		0.279
Technical ratio	1	2	1.13	0.404	0.247
Combined ratio	2	3	1.83	0.657	0.152
Expense ratio	3	4	2.75	0.985	0.102
Loss ratio	4	5	3.67	1.313	0.076
Share market	5	6	4.58	1.642	0.061
Premium growth	6	7	5.5	1.970	0.051
No. of business lines	7	8	8.8	3.152	0.032

On inspection of Table 6, we conclude that in the process of weighting the criteria for ranking Spanish non-life insurance companies, the most important criterion for the expert, ROE, has a weight of 0.279 and the worst, number of business lines, 0.032.

#### Multiple reference point to calculate the global scores of the non-life insurance companies.

Phase 2 of the process requires setting the reference levels. We have used three reference points $(n = 3)$ for each criterion established according to both statistical values and expert knowledge. Specifically, the reference levels corresponding to *Premium Growth*, *ROE*, *Technical Ratio* and *Number of Business Lines* are obtained according to the three first quartiles of the data distributions (see Tables 7, 8, 9,10). For the criteria of the *Loss Ratio*, *Expense Ratio* and *Combined Ratio,* reference levels were set by the DM according to the values displayed in Table 11.

**Table 7:** Reference levels for the *premium growth*

Year	$q^{0} = min$	$q^{1} = Q_{1}$	$q^{2} = Q_{2}$	$q^{3} = Q_{3}$	$q^{4} = max$
2009	− 0.79	− 0.0398	0.0045	0.0468	5.2892
2010	− 0.21	− 0.0203	0.0207	0.0495	6.8754
2011	− 0.35	− 0.0211	0.0133	0.0545	1.0499
2012	− 0.22	− 0.0388	0.0072	0.0535	0.4837
2013	− 0.24	− 0.0435	0.0023	0.0426	1.4128
2014	− 0.21	− 0.0473	− 0.0067	0.0267	0.5499
2015	− 0.40	− 0.0352	0.0091	0.0440	1.0667
2016	− 0.99	− 0.0176	0.0262	0.0740	0.3819
2017	− 0.28	− 0.0112	0.0232	0.0638	0.2869

**Table 8:** Reference levels for the *ROE*

Year	$q^{0} = min$	$q^{1} = Q_{1}$	$q^{2} = Q_{2}$	$q^{3} = Q_{3}$	$q^{4} = max$
2009	− 0.504	0.027	0.086	0.167	0.671
2010	− 2.934	0.015	0.081	0.167	0.632
2011	− 0.598	0.032	0.103	0.198	0.631
2012	− 0.481	0.024	0.103	0.201	0.658
2013	− 0.276	0.028	0.097	0.168	0.572
2014	− 0.272	0.032	0.092	0.173	0.555
2015	− 0.188	0.030	0.095	0.166	0.529
2016	− 0.260	0.034	0.092	0.169	0.518
2017	− 0.301	0.041	0.084	0.1647	0.631

**Table 9:** Reference levels for the *technical ratio*

Year	$q^{0} = min$	$q^{1} = Q_{1}$	$q^{2} = Q_{2}$	$q^{3} = Q_{3}$	$q^{4} = max$
2009	− 0.179	0.030	0.078	0.135	0.585
2010	− 0.282	0.0ç18	0.075	0.149	0.533
2011	− 0.492	0.041	0.097	0.163	0.486
2012	− 0.362	0.036	0.083	0.169	0.685
2013	− 0.230	0.033	0.087	0.184	0.552
2014	− 0.548	0.035	0.077	0.168	0.706
2015	− 0.383	0.031	0.074	0.153	0.730
2016	− 0.228	0.034	0.086	0.160	0.601
2017	− 1.359	0.041	0.089	0.163	0.629

**Table 10:** Reference levels for the *number of business lines*

Year	$q^{0} = min$	$q^{1} = Q_{1}$	$q^{2} = Q_{2}$	$q^{3} = Q_{3}$	$q^{4} = max$
2009	1	2	3	8	18
2010	1	2	3.5	8	19
2011	1	2	4	8	19
2012	1	2	4	8	19
2013	1	2	4	8	19
2014	1	2	4	8.75	19
2015	1	2	4	9	19
2016	1	2	4	8	19
2017	1	2	4	8	19

**Table 11:** Reference levels set by the DM for all years

	$q^{0}$	$q^{1}$	$q^{2}$	$q^{3}$
Loss ratio	0	0.60	0.800	1.0000
Expense ratio	0	0.20	0.400	1.0000
Combined ratio	0	0.80	1.000	1.2000

## Results

The global scores obtained by applying the proposed EBW-MRP(*WS*) model to our database, according to the composite measure, $WS_{i} ,$ proposed in (19), are presented in Table A2 of the Appendix. We have applied the Jarque Bera test for the nine series and normality cannot be rejected at the 5% significance level for all of them. Table 12 summarises the distribution of the EBW-MRP(*WS*) scores for the four tranches. Few companies achieve good results simultaneously

for the eight criteria analysed. The same happens in the case of companies with poor results in all criteria This shows the conflict that exists between the different criteria that measure the comprehensive performance of the companies. For all the years the upper intermediate tranche includes more companies than the lower. Moreover, in most years there are more companies in the upper tranche than in the lower one. Therefore, we can point out a good performance of the Spanish non-life insurance market during the analysed period.

**Table 12:** Number of companies in each score tranche

Score/Year	2009	2010	2011	2012	2013	2014	2015	2016	2017
[3,4]	1	3	5	5	6	3	7	6	7
[2,3]	45	43	42	39	39	46	39	44	43
[1,2]	31	33	32	33	35	30	36	30	30
[0,1]	3	1	2	4	1	3	1	3	3

According to Table 13, sixteen firms remain above the mean of the EBW-MRP(*WS*) scores for all the periods analysed (from 2009 to 2017). On the other hand, fourteen companies remain below the corresponding means. From Table 13, we conclude that mutual insurers rank worse than stock companies, as only one mutuality always appears above the mean (out of 16 firms); while 7 of the 14 insurers that are always below the mean are mutual ones. The hypothesis that the proportion of mutual insurance companies above the score mean is lower than the same proportion for the stock companies is accepted at the 5% significance level (*p*-value = 0.03408). In addition, the hypothesis that the proportion of mutual insurers always below the score mean is greater than the same proportion for the stock companies is accepted at the 5% significance level (*p*-value = 0.04306). It means that, according to the EBW-MRP(*WS*) score, stocks firms perform better than mutual insurers, confirming the results of previous research (Cummins and Nini 2002; Gagarnis et al., 2015).

**Table 13:** Firms above and below the mean

FIRMS ABOVE THE EBW-MRP(WS) MEAN FOR ALL YEARS	FIRMS BELLOW THE EBW-MRP(WS) MEAN FOR ALL YEARS
LA_FE	APOCALIPSIS
ERGO	MARTIERRA
SEGURCAIXA	U_ALCOYANA
PREV_BILBAINA	AME
UNION_MEDICA	U_MADRILEÑA
HERCULES	IMQ_COLEGIAL
SANITAS	PREV_ESPAÑOLA
ASIST_CLINICA	MMT*
EXPERTIA	MUSSAP*
ACUNSA	MURIMAR*
EUROP	AGROMUTUA*
LINEA_DIRECTA	SOCIED_FILANT*
NAT_NEDERL	EL_VOLANTE*
IMQ_SEG_REASEG	UNION_SANIT*
BANSABADELL	
SERAS*	

^*^Mutual insurance company

**Fig. 4:**
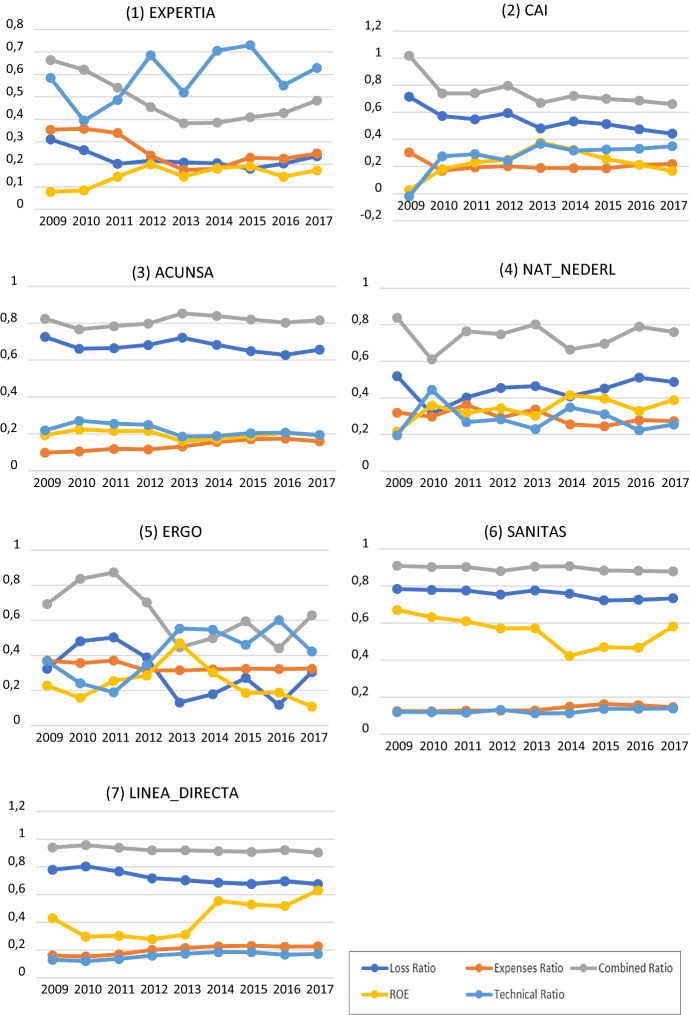
Financial ratios for the seven insurers that score in the top 10 at least 5 times

Table A3 in the Appendix displays the top 10 insurers for each year according to our scoring. For 2010, 2012, 2014, 2015, 2016 and 2017 years it is possible to accept at the 5% significance level the hypothesis that the proportion of mutual insurers is less than the proportion of stock companies in the top ten of the best firms. We focus on the 7 insurers that score in this top 10 at least 5 times, their main financial ratios appearing in Fig. 4. Undoubtedly, EXPERTIA appears as the best firm during all the observed period, ranking first for 5 years. Surprisingly, it is not one of the largest insurers and represented only 0.015% of the market share in 2017, because it only operates in two business lines -assistance and death insurance-. Nevertheless, being a smaller insurer is not a factor which impairs efficiency, profitability or an overall good performance. Looking at the financial health of EXPERTIA, we observe an important decrease of the Combined Ratio over the whole period due to an improvement in both the loss and the expenses ratios. This reveals an enhancement of the insurer’s ability to manage risk and daily activity, which can also be seen in the positive evolution of its ROE.

The second-best firm is CAI, ranking 8 out of 9 years amongst the 10 best firms according to our scores. It had a market share of 0.048% in 2017 despite operating in up to 7 lines of business. As can be seen in Fig. 4.2, CAI experienced an improvement in its financial health since 2009. It diminished its Combined Ratio and improved its ROE and Technical Ratio. This indicates an important improvement in the capacity of the insurer to generate profits efficiently.

ACUNSA only operates in 2 lines of business, related with health insurance. It has a 0.39% of the market share. The behaviour of the financial ratios for ACUNSA is stable over the whole period and reveals a good performance and efficient management. The high value of the loss ratio is noteworthy, probably because the coverage provided is very costly (it is the Navarra Clinic insurance company, an expert in cancer and experimental treatments). Despite the latter, all the financial ratios of this insurer are close to the expected values for a financially healthy firm.

National-Nederlanden is the Spanish Non-life division of the international company of the same name. In our sample it represents 0.174% of the market share and operates in up to 8 lines of business. Its financial health is good throughout the period. It should be noted that the ROE practically doubles its value, but the Technical Ratio, although it increases, does not do so to the same extent, indicating that a significant part of the company's profit does not come from the underwriting activity. In addition, there is an increase in the Loss Ratio from 2010 onwards which, although it always remains at very acceptable levels, indicates that the company could improve its risk management.

ERGO is the most important insurer in travel insurance, operating in 7 lines with 0.066% of market share in our sample. Although its financial health is good, its behaviour is unstable over time. Until 2013 all ratios improved, although from that moment on the trend changed and the ratios showed a worse performance, mainly derived from an increase in claims. There is also a drop in the ROE, greater than that for the Technical Ratio, which indicates a decrease in the earnings from non-underwriting activity.

Sanitas has the largest market share of those companies appearing at least 5 times in the top 10, with 9.785%. Its insurance activity is focused on 4 health-related business lines. Its financial health is good, and the evolution of its ratios is similar to that of ACUNSA (a company also focused on the health business), with a high value for the Loss Ratio. It should be noted that Sanitas, together with Línea Directa, is the one with the best ROE, standing at around 6%.

Línea Directa also appears 5 times in the top 10 and is the second by market share with 5.924%. It is the most diversified company, operating in up to 10 business lines with differing coverage: auto, home, health, etcetera. Although its financial performance has always been good, as of 2014 there has been a significant increase in the ROE reaching in 2017 a value higher than 0.6%. Furthermore, throughout the period an improvement in risk management translates itself into a decrease in the loss ratio and, consequently, in the Combined Ratio.

To sum up, the seven insurers that appear 5 or more times in our top 10, perform well and present good financial health, being efficient in terms of their business management and profitability. None of them is a mutuality, which reinforces the idea that mutual insurers perform worse than stock insurers. Finally, it seems that market share, and size is not necessary to guarantee a good performance, as most of the firms in this top 10 are not the biggest or those with the largest market share (see Table 14).

**Table 14:** Correlation Coefficients of Market Share and EBW-MRP(*WS*) Rankings

	2009	2010	2011	2012	2013	2014	2015	2016	2017
Spearman	0.067	− 0.237	0.122	0.045	0.164	0.110	0.073	0.082	0.103
Kendall	0.053	− 0.147	0.076	0.021	0.107	0.071	0.057	0.060	0.040

**Fig. 5:**
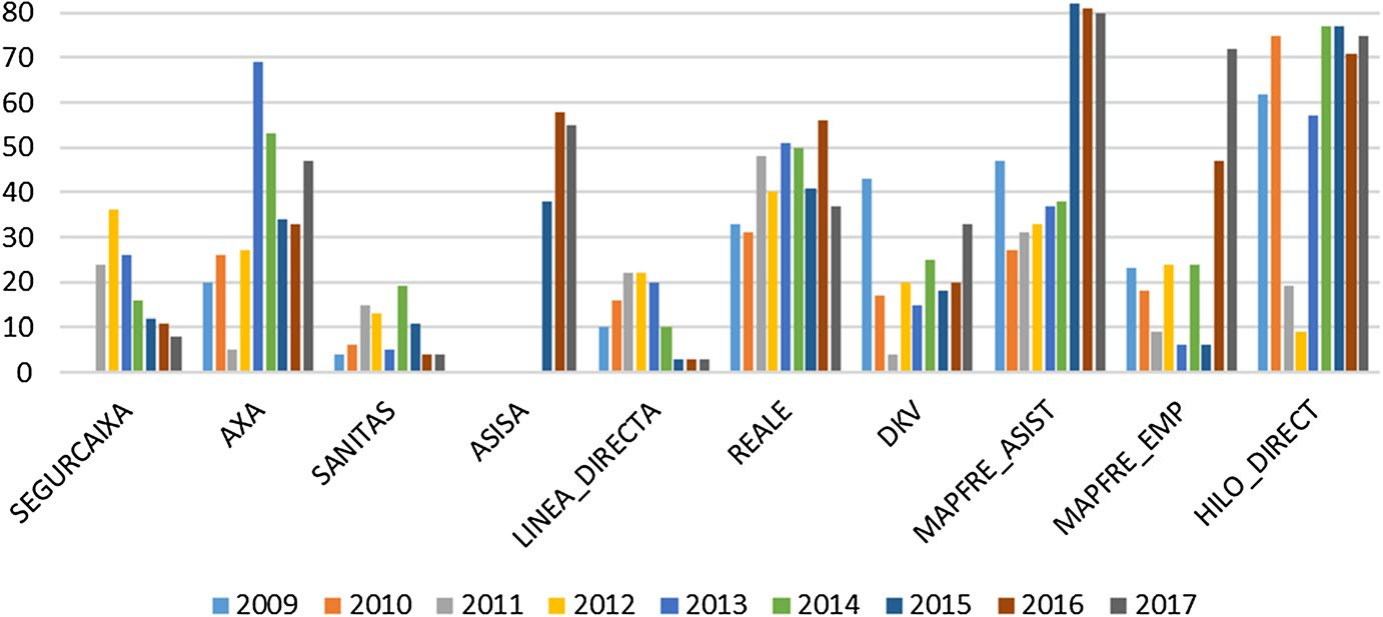
EBW-MRP(WS) rankings for the most important firms by share market

Table 15 and Fig. 5 show the most important firms by share market for our sample. Even those insurers usually large firms, with important amounts of gross premium, are not necessarily ranked as the best ones. These insurers usually operate in up to 13 lines of business, so they are very diversified. Nevertheless, diversification does not indicate good performance or greater profit. In fact, as Cummins and Xie (2013) conclude: “the benefits of diversification come at a cost”. González-Fernández et al (2020), also argue that while diversification allows earning more profits, reducing risk, achieving scope economies and providing higher revenues due to market size, diversification is also related to large and more complex organizations, that incur more management and underwriting costs, which can have a negative impact on profit and performance.

**Table 15:** Score and ranking of the firms accounting for 80% of the market share in Spain

	2009		2010		2011		2012		2013	
FIRM	SCORE	RANK	SCORE	RANK	SCORE	RANK	SCORE	RANK	SCORE	RANK
SEGURCAIXA					2.5654	24	2.3103	36	2.5227	26
AXA	2.5883	20	2.4534	26	3.0009	5	2.5252	27	1.3734	69
SANITAS	2.8882	4	2.8168	6	2.7318	15	2.7992	13	3.0039	5
ASISA										
LINEA_DIRECTA	2.7620	10	2.5950	16	2.5764	22	2.6147	22	2.6693	20
REALE	2.2961	33	2.2419	31	1.9918	48	2.1595	40	1.8555	51
DKV	2.0330	43	2.5555	17	3.0439	4	2.6574	20	2.7822	15
MAPFRE_ASIST	1.9929	47	2.3473	27	2.4301	31	2.3876	33	2.2462	37
MAPFRE_EMP	2.5454	23	2.5306	18	2.7898	9	2.5597	24	3.0030	6
HILO_DIRECT	1.5565	62	1.1976	75	2.6061	19	2.8205	9	1.7409	57
MAX SCORE	3.0673		3.3168		3.2278		3.3444		3.3859	
	2014	2015	2016	2017	TOTAL (MEAN)					
FIRM	SCORE	RANK	SCORE	RANK	SCORE	RANK	SCORE	RANK	SCORE	RANK
SEGURCAIXA	2.7470	16	2.8152	12	2.8168	11	2.9569	8	2.6763	12
AXA	1.9528	53	2.3278	34	2.3838	33	2.1264	47	2.3036	33
SANITAS	2.6391	19	2.8180	11	3.0638	4	3.0863	4	2.8719	5
ASISA			2.1837	38	1.8574	58	1.8808	55	1.9740	50
LINEA_DIRECTA	2.9285	10	3.2033	3	3.2000	3	3.2352	3	2.8649	6
REALE	1.9974	50	2.1550	41	1.9475	56	2.3449	37	2.1100	46
DKV	2.5464	25	2.6780	18	2.6079	20	2.3952	33	2.5888	16
MAPFRE_ASIST	2.2214	38	1.0685	82	0.9372	81	1.0416	80	1.8525	56
MAPFRE_EMP	2.5533	24	3.0105	6	2.1220	47	1.4840	72	2.5109	19
HILO_DIRECT	1.2561	77	1.2655	77	1.5157	71	1.2112	75	1.6856	64
MAX SCORE	3.3947		3.3824		3.2480		3.3636		3.2157

Kendall’s (tau) and Spearman’s (rho) rank correlation coefficients are calculated in order to analyse the relation between company rankings in different years. We have chosen for comparison those pairs of years in which the analysed insurers coincided. As observed in Table 16, a meaningful relation between the performance rankings is revealed with the latter analysis. There is a significant correlation between the performance rankings obtained through the EBW-MRP(*WS*) method for the different years. This shows that the resulting rankings are close to each other for each pair of years compared.

**Table 16:** Spearman and Kendall rank correlation results

	2009–2010	2011–2012	2011–2013	2012–2013	2015–2016	2015–2017	2016–2017
Spearman	0.7120	0.8168	0.7247	0.8263	0.7378	0.6764	0.7593
Kendall	0.5494	0.6346	0.5611	0.6438	0.5810	0.5110	0.5880

Table 17 shows the ranking of the top 10 insurers, for the year 2017, according to the different composite measures, $WS_{i}$, $SS_{i}$ and $PS_{i}$, proposed in (19), (22) and (23), respectively. The last three columns of Table 17 correspond to the partial compensatory measure when we consider three different thresholds 0.65, 0.55 and 0.3. EXPERTIA appears as the best firm when applying the weighted mean $WS_{i}$, but surprisingly this firms is not in the top 10 when applying the strong composite measure $SS_{i}$. This is due to EXPERTIA being a non-diversified insurer with a very low value for the number of business lines criterion. When we consider a partial compensatory measure, this firm is not so penalized and again ranks in the top 10. Table 18 shows the Spearman and Kendall correlation coefficients between the insurers’ rankings obtained for the different composite measures. There are high and significant correlations between the rankings obtained through the weighted mean $WS_{i}$ and the partial compensatory measures $PS_{i}$ for all the thresholds considered. When the relationship between $SS_{i}$ and $WS_{i}$ is analysed, the Spearman and Kendall correlation coefficients are lower. The top 10 insurers prove different for both measures. In addition to EXPERTIA, another three insurers disappear for the $SS_{i}$ ranking. We also observe that when applying the $PS_{i}$ measure, the correlation with the $WS_{i}$ measure rises as does the threshold considered.

**Table 17:** Top 10 best firms according to the different composite measures for 2017

EBW-MRP ($WS_{i}$)	EBW-MRP ($SS_{i}$)	EBW-MRP ($PS_{i}$(0.65))	EBW-MRP ($PS_{i}$(0.55))	EBW-MRP ($PS_{i}$(0.3))
EXPERTIA	NAT_NEDERL	NAT_NEDERL	NAT_NEDERL	LINEA_DIRECTA
NAT_NEDERL	LINEA_DIRECTA	LINEA_DIRECTA	LINEA_DIRECTA	NAT_NEDERL
LINEA_DIRECTA	MERIDIANO	EXPERTIA	EXPERTIA	SANITAS
SANITAS	SEGURCAIXA	MERIDIANO	MERIDIANO	PREV_BILBAINA
MERIDIANO	SANITAS	SANITAS	SANITAS	MERIDIANO
PREV_BILBAINA	LA_FE	SEGURCAIXA	PREV_BILBAINA	SEGURCAIXA
ACUNSA	AURA	PREV_BILBAINA	SEGURCAIXA	EXPERTIA
SEGURCAIXA	DAS	AURA	ACUNSA	AURA
CAI	ASIST_SANIT	IMQ_NAVARRA	IMQ_NAVARRA	IMQ_NAVARRA
AURA	IMQ_NAVARRA	ACUNSA	AURA	LA_FE

**Table 18:** Spearman and Kendall rank correlation results obtained by different composite measures

	*WS* vs *SS*	*WS* vs *PS* (0.65)	*WS* vs *PS* (0.55)	*WS* vs *PS* (0.3)	*SS* vs *PS* (0.65)	*SS* vs *PS* (0.55)	*SS* vs *PS* (0.3)
Spearman	0.670	0.991	0.988	0.964	0.703	0.681	0.694
Kendall	0.531	0.931	0.918	0.845	0.572	0.555	0.569

### Sensitivity analysis

The sensitivity of the EBW-MRP(*WS*) model is analysed via changes in several parameters. We have modified the interval comparisons between pairs of criteria in line with Table 19. Here, keeping ROE as the most important criterion, the Loss Ratio is the second most important criterion, followed by the Technical and Combined Ratios. The resulting weights (last column in Table 19) reflect these preferences. In addition, the parameter $d_{W}$ has been set equal to 0.05. The value achieved by $\lambda^{*}$ is, in this case, equal to 0.641, therefore achieving consistency within the extended intervals. The Spearman correlation coefficient between the scores of the original and disturbed models is equal to 0.977 (Kendall coefficient equal to 0.887). The top ten companies remain for both rankings except La Fe which goes up five places and ranks among the top ten in the disturbed model.

**Table 19:** Pairwise preferential ratios

Criteria	$l_{Bj}$	$u_{Bj}$	$l_{jW}$	$u_{jW}$	Weights
ROE	1	1	8	9	0.2513
Technical ratio	2	3	6	7	0.1436
Combined ratio	2	3	6	7	0.1436
Expense ratio	3	4	4	5	0.0958
Loss ratio	1	2	7	8	0.2334
Share market	5	6	2	3	0.0574
Premium growth	6	7	1	2	0.0479
No. of business lines	7	8	1	1	0.027

The other sensitivity exercise carried out consisted in using the reference points arising from the statistical values (the three first quartiles), for all criteria. In this situation, the Spearman correlation between the scores of the original and disturbed models is equal to 0.99, therefore signifying that results are highly correlated.

## Conclusions

The paper proposes a model for ranking non-life insurance companies based on combining the most relevant financial ratios and indicators into one overall score, thereby measuring the composite performance of each firm.

We have attempted to resolve two issues associated with such scoring approaches that may preclude decision making. The pairwise comparisons are useful tools for identifying the relative importance between the decision elements. The study takes advantage of this suitability, well referenced in the literature, and proposes an approach for overcoming two ongoing problems, such as the imprecision in judgements and the difficulty for making many paired comparisons. The first issue is addressed by interval values that are extended by tolerance thresholds. The second one is overcome by restricting paired comparisons to those where one element of the pair is the most (least) important of the decision criteria. This approach extends the Best–Worst method and is addressed by fuzzy set theory. The aggregation procedure chosen uses both knowledge expert and statistical values, facilitating a good balance between objective and subjective information. In addition, the MRP methodology gives global scores within the scale set by the DM making them meaningful for the DM. The proposal is comfortable for the DM because it is based on easily obtainable expert knowledge as well as easily understandable results. Mathematically, the proposal is within a linear framework.

Several findings are obtained from the implementation of the model for a Spanish database spanning the years 2009 to 2017. The distribution of the score obtained allows us to conclude that the Spanish non-life insurance market performance is reasonably good given that most of the companies are located in the upper tranches.

The results reveal, as expected, that stock (private) companies perform better and are more profitable than mutual companies. At the same time, small companies score higher than large ones, revealing that size and diversification does not offer a real advantage. Maybe management inefficiencies and the increase in cost make large firms underperform when compared with smaller firms. Nevertheless, the financial study made for the Spanish non-life insurance market, allows us to affirm that it benefits from good financial health, based on the financial ratios analysed. Although over the whole period premium growth was positive, the financial crisis caused a slowdown in the latter from 2010 onwards. The impact of the financial crisis is also observed via the decrease of the ROE and technical ratio in 2010, although these ratios recuperate during the period analysed, albeit not reaching their previous values. The positive evolution of these ratios as well as the Combined Ratio reveals a favourable trend for the Spanish non-life insurance market.

Our method is easily comprehensible and includes both hard and soft data. We hope that these features will give rise to a widespread use by practitioners. In terms of future research, the proposed method may be extended to include group decision-making involving more than one DM. In addition, the EBW method could be combined with other scoring methodologies. We also suggest applying the proposed method to other markets. In the context of the COVID-19 pandemic it is important to check how have changed the financial performance of the insurance companies. The future research could consider the impact of the COVID-19 pandemic on the global scores to find the correlation between periods before and after the pandemic.

## Appendix

See Tables 20, 21, 22.

**Table 20:** Insurance companies

COMPANIES	ABREV
C1. AGROMUTUA-MAVDA S.M.S.P.F	AGROMUTUA
C2. AGRUPACION SANITARIA SEGUROS S.A	AGRUP_SANIT
C3. ALMUDENA CIA DE SEGUROS Y REASEGUROS S.A	ALMUDENA
C4. AME ASISTENCIA MÉDICA CÍA SEGUROS S.A	AME
C5. AMSYR AGRUPACIO SEGUROS Y REASEGUROS, S.A	AMSYR
C6. APOCALIPSIS FUNERARIOS REUNIDOS S.A	APOCALIPSIS
C6. ARLI MUTUALIDAD DE PREVISON DE LAS ARTES DLE LIBRO	ARLI
C8. ASEMAS MUTUA SEGUROS Y REASEGUROS A PRIMA FIJA	ASEMAS
C9. ASISA, ASISTENCIA SANITARIA INTERPROVINCIAL DE SEGUROS S.A	ASISA
C10. ASISTENCIA CLINICA UNIVERSITARIA DE NAVARRA S.A. DE SEGUROS Y@ REASEGUROS	ACUNSA
C11. ASISTENCIA SANITARIA COLEGIAL	ASIST_SANIT
C12. ASOCIACION EUROPEA CIA. DE SEGUROS, S.A	ASOC_EUROPEA
C13. ATLANTIDA MEDICA DE ESPECIALIDADES, S.A	ATLANTIDA
C14. ATOCHA S.A. DE SEGUROS	ATOCHA
C15. AURA SA DE SEGUROS	AURA
C16. AXA SEGUROS GENERALES, SA DE SEGUROS Y REASEGUROS	AXA
C17. BANSABADELL SEGUROS GENERALES, S.A	BANSABADELL
C18. CAI SEGUROS GENERALES	CAI
C19. CASER MEDITERRANEO SEGUROS GENERALES S.A	CASER
C20. CENTRO DE PROTECCION DE CHOFERES DE LA RIOJA	CENTRO_RIOJA
C21. CIA DE SEG IGUALATORIO MEDICO QUIRURGICO Y DE ESPECIALIDADES DE @ NAVARRA	IMQ_NAVARRA
C22. CLINICUM SEGUROS SA	CLINICUM
C23. COMPAÑIA DE SEGUROS PREVISION MEDICA, S.A	COMP_SEGUROS
C24. COMPAÑIA ESPAÑOLA DE SEGUROS DE CREDITO A LA EXPORTACION, CESCE SA	CESCE
C25. DAS DEFENSA DEL AUTOMOVILISTA Y DE SINIESTROS INTERNACIONAL SA DE@ SEGUROS Y REASEGUROS	DAS
C26. DIVINA PASTORA SEGUROS GENERALES S.A	DIVINA
C27. DKV SEGUROS Y REASEGUROS S.A.E	DKV
C28. EL VOLANTE ARAGONES, MONTEPIO DE CONDUCTORES	EL_VOLANTE
C29. ERGO GENERALES SEGUROS Y REASEGUROS	ERGO
C30. ETERNA ASEGURADORA, S.A	ETERNA
C31. EUROP ASSISTANCE ESPAÑA, S.A. DE SEGUROS Y REASEGUROS	EUROP
C32. EXPERTIA SEGUROS DE DECESOS S.A	EXPERTIA
C33. FENIX DIRECTO COMPAÑIA DE SEGUROS Y REASEGUROS S.A	FENIX
C34. HERCULES SALUD SEGUROS, S.A	HERCULES
C35. HILO DIRECT SEGUROS, SA	HILO_DIRECT
C36. IGUALATORIO DE PREVISION SANITARIA S.A. DE SEGUROS	IGUAL_PREVISION
C37. IGUALATORIO MEDICO QUIRURGICO Y DE ESPECIALIDADES DE ASTURIAS, S.A.@ DE SEGUROS	IMQ_ASTURIAS
C38. IGUALATORIO MEDICO QUIRURGICO, S.A., DE SEGUROS Y REASEGUROS	IMQ_SEG_REASEG
C38. IGUALATORIO MEDICO-QUIRURGICO COLEGIAL, S. A. DE SEGUROS	IMQ_COLEGIAL
C40. IMA IBERICA SEGUROS Y REASEGUROS, S.A	IMA
C41. LA FE PREVISORA COMPAÑIA DE SEGUROS, S.A	LA_FE
C42. LA PREVISION MALLORQUINA DE SEGUROS, S.A	LA_PREVISIÓN
C43. LA UNION ALCOYANA, S.A. DE SEGUROS Y REASEGUROS	U_ALCOYANA
C44. LA UNION MADRILEÑA DE SEGUROS S.A	U_MADRILEÑA
C45. LINEA DIRECTA ASEGURADORA, S.A. CIA DE SEGUROS Y REASEGUROS	LINEA_DIRECTA
C46. MAPFRE ASISTENCIA COMPAÑIA INTERNACIONAL DE SEGUROS Y REASEGUROS,@ S.A	MAPFRE_ASIST
C47. MAPFRE EMPRESAS, CIA. DE SEGUROS Y REASEGUROS, S.A	MAPFRE_EMP
C48. MARTIERRA SEGUROS, S.A	MARTIERRA
C49. MERIDIANO, S.A. CIA. ESPAÑOLA DE SEGUROS	MERIDIANO
C50. MUSAAT, MUTUA DE SEGUROS A PRIMA FIJA	MUSAAT
C51. MUSSAP MUTUA DE SEGUROS Y REASEGUROS A PRIMA FIJA	MUSSAP
C52. MUTUA DE PROPIETARIOS, SEGUROS Y REASEGUROS, APF	MUTUA_PROPIET
C53. MUTUA DE RIESGO MARITIMO, SOCIEDAD SEGUROS A PRIMA FIJA (MURIMAR)	MURIMAR
C54. MUTUA MMT SEGUROS	MMT
C55. MUTUA TINERFEÑA, MUTUA DE SEGUROS Y REASEGUROS A PRIMA FIJA	MUTUA_TINERF
C56. MUTUALIDAD ARROCERA DE SEGUROS	MUTUAL_ARROCERA
C57. MUTUALIDAD DE LEVANTE ENTIDAD DE SEGUROS A PRIMA FIJA	MUTUAL_LEVANTE
C58. MUTUALIDAD DE PREVISION SOCIAL DE FUTBOLISTAS ESPAÑOLES	MUTUAL_FUTBOL
C59. MUTUASPORT, MUTUA DE SEGUROS DEPORTIVOS A PRIMA FIJA	MUTUASPORT
C60. MUTUAVENIR MUTUA DE S.R.P.F. DE PAMPLONA	MUTUAVENIR
C61. NATIONALE-NEDERLANDEN GENERALES CIA. DE SEGUROS Y REASEGUROS, @ S.A.E	NAT_NEDERL
C62. P.S.N. AGRUPACION MUTUAL ASEGURADORA (AMA)	AMA
C63. POLICLINICO CENTRO MEDICO DE SEGUROS, SA	POLICLINICO
C64. PREVISORA BILBAINA SEGUROS, S.A	PREV_BILBAINA
C65. PREVISORA ESPAÑOLA DE ESPECIALIDADES Y SEGUROS SA	PREV_ESPAÑOLA
C66. REALE SEGUROS GENERALES, S.A	REALE
C67. S SEG MUTUOS INCENDIOS EDIFICIOS RURALES ALAVA	S_SEG_INCEND
C68. SABADELL ASEGURADORA, SA	SABADELL
C69. SALUS ASISTENCIA SANITARIA S.A. DE SEGUROS	SALUS
C70. SANITAS SOCIEDAD ANONIMA DE SEGUROS	SANITAS
C71. SEGURCAIXA, S.A. DE SEGUROS Y REASEGUROS	SEGURCAIXA
C72. SEGUROS GENERALES RURAL, S.A	SEGUROS_GEN_RURAL
C73. SEGUROS LAGUN ARO, S.A	LAGUN_ARO
C74. SERAS MUTUALIDAD DE SEGUROS A PRIMA FIJA	SERAS
C75. SOCIEDAD FILANTROPICA DEL COMERCIO, INDUSTRIA Y BANCA DE MADRID	SOCIED_FILANT
C76. SOCIEDAD SEGUROS MUTUOS CONTRA INCENDIOS DE VALENCIA	SOCIED_SEGUR_MUTUOS
C77. SOLISS MUTUALIDAD DE SEGUROS Y REASEGUROS A PRIMA FIJA	SOLISS
C78. SOS SEGUROS Y REASEGUROS, S.A	SOS
C79. UNION DE AUTOMOVILES CLUBS SA DE SEGUROS Y REASEGUROS	UNION_AUTO
C80. UNION MEDICA LA FUENCISLA, S.A	UM_FUENCISLA
C81. UNION SANITARIA MEDICO QUIRURGICA	UNION_SANIT
C82. UNIVERSAL ASISTENCIA DE SEGUROS Y REASEGUROS, S.A	UNIVERSAL
C83. VITAL SEGURO S.A	VITAL

**Table 21:** EBW-MRP(*WS*) scores for all the insurance companies

COMPANY	2009	2010	2011	2012	2013	2014	2015	2016	2017	MEAN
C1	1.326	1.456	1.499	1.748	1.702	1.456	1.284	1.493	0.959	1.436
C2	3.067	2.718	2.763	2.782	3.042	3.046	3.113	2.809	2.752	2.899
C3	2.649	2.476	2.478	2.442	2.805	2.734	2.335	2.487	2.123	2.503
C4	1.280	1.447	1.355	1.594	1.583	1.386	1.628	1.358	1.726	1.484
C5	2.574	1.955	2.474	2.275	2.474	2.151	1.940	1.840	1.779	2.163
C6	0.987	1.309	1.375	1.715	1.538	1.702	1.657	1.707	1.743	1.526
C7	2.025	2.093	2.444	1.962	1.831	1.623	2.084	1.949	1.509	1.947
C8	1.122	1.136	1.434	0.978	1.142	1.508	1.923	2.516	2.489	1.583
C9							2.184	1.857	1.881	1.974
C10	2.717	2.795	2.791	2.797	2.575	2.968	3.007	3.061	3.010	2.858
C11	2.032	1.974	1.522	1.719	2.323	2.010	2.124	1.969	2.536	2.023
C12	2.076	2.226	2.342	2.412	2.224	1.810	1.679	2.336	2.515	2.180
C13	1.780	2.101	1.761	1.629	1.821	2.337	2.015	1.706	1.626	1.864
C14	2.774	2.779	2.778	2.502	2.993	2.992	2.177	2.065	2.103	2.574
C15	1.696	2.327	2.128	2.882	2.651	2.998	2.236	2.910	2.825	2.517
C16	2.588	2.453	3.001	2.525	1.373	1.953	2.328	2.384	2.126	2.304
C17	2.564	2.203	2.862	2.952	2.892	2.542	2.526	2.839	2.447	2.647
C18	1.742	3.206	3.228	3.177	3.386	2.950	2.901	2.870	2.839	2.922
C19	2.221	1.949	2.517	3.228	2.670	2.781	2.813	2.921	2.407	2.612
C20	2.318	2.237	1.904	1.954	1.884	1.780	1.220	1.012	1.600	1.768
C21	2.032	2.486	1.560	1.768	2.513	2.113	2.158	2.298	2.802	2.192
C22	1.396	1.387	1.485	1.849	1.823	1.825	2.561	2.360	2.190	1.875
C23	1.375	1.404	1.399	1.378	1.293	1.755	2.153	2.228	2.361	1.705
C24	1.907	3.317	2.236	1.763	2.176	2.485	1.937	1.858	1.889	2.174
C25	0.900	1.240	1.402	1.410	1.718	1.671	1.770	2.480	2.664	1.695
C26	1.798	0.998	1.599	2.106	2.734	2.362	3.095	2.056	2.783	2.170
C27	2.033	2.556	3.044	2.657	2.782	2.546	2.678	2.608	2.395	2.589
C28	2.068	1.787	1.800	1.934	1.647	1.753	1.703	0.925	0.952	1.619
C29	2.929	2.641	2.634	2.803	3.199	2.985	2.856	3.033	2.637	2.858
C30	2.345	2.507	2.476	2.479	2.271	2.005	1.691	1.705	2.655	2.237
C31	2.434	2.296	2.327	2.814	2.384	2.243	2.501	2.461	2.431	2.432
C32	2.893	2.893	3.144	3.344	3.279	3.395	3.382	3.248	3.364	3.216
C33	1.573	2.214	1.337	1.251	1.090	1.591	1.631	1.031	1.198	1.435
C34	2.705	2.779	2.714	2.720	2.790	2.740	2.732	2.773	2.667	2.736
C35	1.556	1.198	2.606	2.821	1.741	1.256	1.265	1.516	1.211	1.686
C36	2.358	2.233	1.859	2.018	2.246	2.579	2.529	2.245	2.272	2.260
C37	2.620	2.242	2.015	1.769	1.991	2.632	1.872	2.234	2.185	2.173
C38	2.506	2.473	2.231	2.552	2.491	2.509	2.598	2.622	2.380	2.485
C39	1.668	1.802	1.714	1.605	1.947	1.879	1.709	1.567	1.688	1.731
C40	2.885	1.303	2.517	1.754	1.358	1.455	1.918	1.509	2.251	1.883
C41	2.831	2.842	2.754	2.903	2.874	2.828	2.725	2.792	2.749	2.811
C42	1.867	1.880	1.480	1.786	2.141	2.121	2.373	2.105	2.686	2.049
C43	1.919	1.318	1.624	1.388	1.898	1.431	1.754	1.808	1.968	1.679
C44	1.372	1.400	1.647	1.598	1.445	1.430	1.543	1.438	1.793	1.518
C45	2.762	2.595	2.576	2.615	2.669	2.928	3.203	3.200	3.235	2.865
C46	1.993	2.347	2.430	2.388	2.246	2.221	1.068	0.937	1.042	1.853
C47	2.545	2.531	2.790	2.560	3.003	2.553	3.011	2.122	1.484	2.511
C48	1.507	1.749	2.014	1.386	1.325	1.635	1.335	1.343	1.640	1.548
C49	2.619	2.596	2.391	2.728	2.249	1.885	2.047	2.195	3.035	2.416
C50	0.906	1.115	1.152	1.473	1.304	2.056	1.901	2.307	1.838	1.561
C51	1.227	1.227	0.882	0.957	0.639	0.261	0.339	0.895	1.138	0.840
C52	1.807	2.207	2.891	2.707	2.884	2.814	2.422	2.609	2.131	2.497
C53	1.226	1.397	1.150	1.345	1.150	0.884	1.268	1.561	1.780	1.307
C54	1.657	1.518	1.367	1.438	1.266	1.259	1.294	1.124	1.464	1.376
C55	2.190	2.080	1.841	1.888	2.028	2.403	1.902	1.975	2.315	2.069
C56	1.183	1.264	1.219	0.909	1.065	1.132	1.201	2.522	1.095	1.288
C57	2.119	1.471	1.790	2.248	1.594	2.237	1.429	1.627	1.963	1.831
C58	1.264	1.311	1.875	1.080	1.650	1.636	2.277	1.989	1.772	1.651
C59	1.538	1.009	0.739	0.422	1.793	2.028	1.793	2.418	2.455	1.577
C60	1.554	2.478	2.781	1.548	1.523	2.156	2.354	2.546	2.493	2.159
C61	2.687	3.025	3.162	3.179	2.823	3.333	3.310	3.208	3.242	3.108
C62	1.857	2.126	2.422	2.811	2.591	2.622	2.508	2.456	2.217	2.401
C63	2.051	1.168	1.689	1.702	1.795	1.611	1.857	2.299	1.719	1.766
C64	2.791	2.742	2.757	2.732	2.688	2.865	2.489	2.407	3.024	2.722
C65	1.634	1.679	1.575	1.279	1.408	0.872	1.614	1.561	1.606	1.470
C66	2.296	2.242	1.992	2.159	1.855	1.997	2.155	1.948	2.345	2.110
C67	2.450	1.483	2.584	2.621	2.096	2.815	1.219	2.574	2.332	2.241
C68						1.962	2.447	2.302	2.095	2.202
C69	2.743	2.236	2.174	1.796	1.724	2.494	2.849	2.695	2.644	2.373
C70	2.888	2.817	2.732	2.799	3.004	2.639	2.818	3.064	3.086	2.872
C71			2.565	2.310	2.523	2.747	2.815	2.817	2.957	2.676
C72	1.756	1.903	1.702	1.993	1.907	2.040	1.837	2.602	1.815	1.950
C73	2.071	2.049	2.386	3.122	2.868	2.630	2.520	2.280	2.405	2.481
C74	2.474	2.517	2.341	2.393	2.364	2.153	2.345	2.298	2.267	2.350
C75	1.514	1.924	1.687	1.872	1.155	1.724	1.948	1.590	1.471	1.654
C76	2.268	2.106	2.185	2.089	2.207	1.626	1.264	1.387	0.562	1.744
C77	2.780	2.222	2.231	2.590	2.566	2.150	1.926	2.421	2.598	2.387
C78	2.706	2.630	2.583	2.103	2.588	2.991	2.766	2.762	2.545	2.631
C79	2.506	1.994	2.573	2.358	2.383	2.357	2.248	2.268	2.487	2.353
C80	2.371	2.112	2.321	2.367	2.704	2.536	2.435	2.762	2.353	2.440
C81	1.430	1.216	1.473	1.465	1.302	1.139	1.719	1.664	1.049	1.384
C82	2.119	2.492	2.251	2.199	2.468	2.339	1.890	1.979	1.900	2.182
C83	2.640	2.683	2.649	2.543	2.155	1.972	2.751	1.708	1.698	2.311

**Table 22:** Top ten best firms

Top ten best firms (2009)	Top ten best firms (2010)
AGRUP_SANIT	COMP_ESPAÑOLA
ERGO	CAI
EXPERTIA	NAT_NEDERL
SANITAS	EXPERTIA
IMA	LA_FE
LA FE	SANITAS
PREV_BILBAINA	ACUNSA
SOLISS*	ATOCHA
ATOCHA	HERCULES
LINEA_DIRECTA	PREV_BILBAINA
**Top ten best firms (2011)**	**Top ten best firms (2012)**
CAI	EXPERTIA
NAT_NEDERL	CASER
EXPERTIA	NAT_NEDERL
DKV	CAI
AXA	LAGUN_ARO
MUTUA_PROPIET*	BANSABADELL
BANSABADELL	LA FE
ACUNSA	AURA
MAPFRE_EMP	HILO_DIRECT
MUTUAVENIR*	EUROP
**Top ten best firms (2013)**	**Top ten best firms (2014)**
CAI	EXPERTIA
EXPERTIA	NAT_NEDERL
ERGO	AGRUP_SANIT
AGRUP_SANIT	AURA
SANITAS	ATOCHA
MAPFRE-EMP	SOS
ATOCHA	ERGO
BANSABADELL	ACUNSA
MUTUA_PROPIET*	CAI
LA FE	LINEA_DIRECTA
**Top ten best firms (2015)**	**Top ten best firms (2016)**
EXPERTIA	EXPERTIA
NAT_NEDERL	NAT_NEDERL
LINEA_DIRECTA	LINEA_DIRECTA
AGRUP_SANIT	SANITAS
DIVINA	ACUNSA
MAPFRE_EMP	ERGO
ACUNSA	CASER
CAI	AURA
ERGO	CAI
SALUS	BANSABADELL
**Top ten best firms (2017)**	
EXPERTIA	
NAT_NEDERL	
LINEA_DIRECTA	
SANITAS	
MERIDIANO	
PREV_BILBAINA	
ACUNSA	
SEGURCAIXA	
CAI	
AURA	

^*^Mutual insurance company

## Footnotes

1 Following Zimmermann (1976), an extended fuzzy linear membership function that expresses the DM’s satisfaction with the accomplishment of the soft constraints $Ax\tilde{ \le }0$ is:

$$\mu (Ax) = 1 - \frac{Ax}{d},\quad {\text{if 0}} \le Ax \le d$$

2 $$m_{jW} = \frac{{\frac{1}{{d_{jW}^{l} }}l_{jW} + \frac{1}{{d_{jW}^{u} }}u_{jW} }}{{\frac{1}{{d_{jW}^{l} }} + \frac{1}{{d_{jW}^{u} }}}}$$

3 See https://www.swissre.com/institute/research/sigma-research/sigma-2018-04.html

4 For reasons of brevity, Table 4 shows the average of the different statistics for the observed period. Individual statistics for each year are available upon request.

5 Without loss of generality, we suppose that all the tolerance parameters are equal, $d_{Bj}^{l} = d_{Bj}^{u} = d_{jW}^{l} = d_{jW}^{u} = d$.

## Declarations

### Confict of interests

The authors declare that they have no confict of interests.
